# Bayesian inference of protein conformational ensembles from limited structural data

**DOI:** 10.1371/journal.pcbi.1006641

**Published:** 2018-12-17

**Authors:** Wojciech Potrzebowski, Jill Trewhella, Ingemar Andre

**Affiliations:** 1 Data Management and Software Centre, European Spallation Source ERIC, Copenhagen, Denmark; 2 Biochemistry and Structural Biology, University of Lund, Lund, Sweden; 3 School of Life and Environmental Sciences, The University of Sydney, Sydney, Australia; University of Maryland School of Pharmacy, UNITED STATES

## Abstract

Many proteins consist of folded domains connected by regions with higher flexibility. The details of the resulting conformational ensemble play a central role in controlling interactions between domains and with binding partners. Small-Angle Scattering (SAS) is well-suited to study the conformational states adopted by proteins in solution. However, analysis is complicated by the limited information content in SAS data and care must be taken to avoid constructing overly complex ensemble models and fitting to noise in the experimental data. To address these challenges, we developed a method based on Bayesian statistics that infers conformational ensembles from a structural library generated by all-atom Monte Carlo simulations. The first stage of the method involves a fast model selection based on variational Bayesian inference that maximizes the model evidence of the selected ensemble. This is followed by a complete Bayesian inference of population weights in the selected ensemble. Experiments with simulated ensembles demonstrate that model evidence is capable of identifying the correct ensemble and that correct number of ensemble members can be recovered up to high level of noise. Using experimental data, we demonstrate how the method can be extended to include data from Nuclear Magnetic Resonance (NMR) and structural energies of conformers extracted from the all-atom energy functions. We show that the data from SAXS, NMR chemical shifts and energies calculated from conformers can work synergistically to improve the definition of the conformational ensemble.

## Author summary

Proteins are commonly built up by folded domains connected by regions with higher flexibility. The interdomain orientations encoded by such hinges or linkers can play central roles in controlling the function of multidomain proteins, which makes them important to characterize. Small Angle X-ray Scattering (SAXS) is uniquely suited to study the conformational ensembles adopted by these kinds of proteins. However, because of the limited information provided by SAXS, ensemble models must be built by combination with other information sources and care have to be taken to avoid constructing ensembles that are more complex than data can support. We developed a method based on Bayesian statistics that combine data from molecular simulation with experimental data from SAXS and Nuclear Magnetic Resonance while automatically balancing the complexity of ensemble model with information in the data. We demonstrate that this method is capable of accurate inference of ensembles even in the presence of high levels of experimental noise. The method represents a general approach to combine data and simulation in the modeling of protein ensembles and can be extended to employ additional sources of experimental information.

## Introduction

Proteins are highly dynamic systems [1] often with large scale conformational dynamics facilitated by regions of flexible or disordered amino acid sequence linking stably folded structured domains [2]. Close to half to the proteins coded in the human genome contain significant disordered regions of greater than 30 residues [3] and there is a multitude of multi-domain proteins with shorter flexible linkers or hinges that are important for their biological function (*e*.*g*.: in enzyme catalysis [4, 5], DNA damage signalling and repair [6], DNA binding and allosteric signalling [7], mechanical properties in the giant protein muscle protein titin [8, 9], target recognition by the intracellular regulatory Ca^2+^-receptor calmodulin [10], and ubiquitin-mediated regulatory mechanisms [11, 12]. These multi-domain proteins connected by flexible regions are difficult to characterize structurally as they tend to be resistant to crystallization, too large for NMR solution structure techniques and often present ambiguous results for microscopy techniques.

The small-angle scattering (SAS) from proteins in solution samples the time and ensemble average of the randomly oriented structures present. For mono-dispersed macro-molecules of uniform size, one can reliably extract accurate structural parameters such as the radius of gyration (*R*_*g*_), molecular weight (*M*), the probability distribution of inter-atomic distances (*P*(*r*) vs. *r*), and an estimate of the molecular volume [13, 14]. Advances with 3D structural modelling against SAS data have further provided more detailed structural interpretation and yielded important biological insights (reviewed in Trewhella et al. [15]). This success has been achieved in spite of the fact that the SAS profile from a protein in solution represents the rotationally averaged 3D structure, hence directional information is lost leaving only 1D distance information that generally can fit multiple 3D solutions. Further, the SAS profile is a smooth function that decays rapidly and can be adequately defined by as few as 10–15 points [16]. When experimental errors are taken into account, the information content is further reduced and it is not uncommon that only 5–10 parameters can be extracted from a SAS profile [17]. Successful 3D modelling against SAS data thus depends upon restraining the conformational space to be sampled by *a priori* knowledge of protein structure and wherever possible by other experimental data.

In the event that a structural ensemble is present, the values of the structural parameters determined and any optimized individual 3D model will represent a population weighted average. Given the abundance of multi-domain proteins with structurally undefined linking sequences, and the difficulty in characterizing them, ensemble or multi-state modelling against SAS data is an increasingly popular choice (see reviews [18–20]. However, the problems arising from the limited information content of the SAS profile are many times amplified with the ensemble model. An ensemble model of 3D structures will have many more degrees of freedom than a single 3D model. As a result, ensemble modelling against a SAS profile is even more vulnerable to over-fitting and over-interpretation, even considering limits to the conformational space to be sampled via restraints such as knowledge of domain structures, specific flexible regions, contact information from NMR, cross-linking or FRET measurements, etc.

The objective of ensemble modelling is to return a set of structural models and their corresponding population weights. Conceptually, we can divide this process into two steps: model selection and weights inference. In model selection we determine the size of the ensemble and which members of the structural library to include. In weight inference the population weights of the selected ensemble is determined. In practice, these steps are often done simultaneously, using minimization of the difference between observed and predicted experimental data as guiding principle (often measured as χ or χ^2^). A number of different approaches has been presented to limit ensemble sizes and overfitting. MultiFoXS [21] optimizes χ for a given number of conformers (usually in the range 1–5) from which a minimal ensemble can be defined. The Sparse Ensemble Selection (SES) method [22] finds an optimal ensemble using linear least squares with a regularization term to obtain a sparse ensemble of conformations. Overfitting can also be combatted by using model comparison metrics like Aikake Information Criteria (AIC), an approach used by Bowerman et al. [23] to select optimal ensembles in their Bayesian ensemble modelling method. For highly flexible systems such as intrinsically disordered proteins, a small number of conformers cannot realistically describe the ensemble. Methods like EOM [24] result in sizable shrinkage of the initial structural library but do not explicitly limit the ensemble size. The use of discrete protein conformations can also be avoided altogether in the modelling of flexible proteins by using a generative probabilistic model of protein structure in Bayesian modelling [25]. A more extensive discussion of approaches for model selection and weight inference is found in the review by Bonomi et al. [26].

Because SAS data does not contain enough information to infer the full ensemble as it is sampled in solution, we choose to find an ensemble that is “optimal” in the sense that it is the simplest model that explains the available experimental data while avoiding fitting to noise. In this study we use model evidence [27] or marginal likelihood, to select ensembles with optimal sets of members. Model evidence (ME) is widely used in Bayesian model comparison and provides an automatic Occam’s razor effect [28] by balancing between fit to data and model complexity, thereby providing a rigorous approach to combat overfitting. However, ME is a multidimensional integral that can be very difficult to evaluate, which is a significant barrier to its use in ensemble selection. Our ensemble selection method is based on an approximate, variational Bayesian inference (VBI) method for model selection pioneered by Fisher and colleagues who used the method to infer ensembles of intrinsically disordered protein from NMR chemical shifts and residual dipolar couplings [29]. The VBI approach has two major benefits. First, it is significantly faster than complete Bayesian inference, which enables the use of large structural libraries. Second, VBI implicitly leads to maximization of ME without the need for evaluation of a multidimensional integral. A downside of the VBI approach is that it involves a few approximations in the probabilistic model. Hence, after arriving at the optimal ensemble with VBI we carry out a complete Bayesian inference of weights which we use to quantify uncertainties in the ensemble model and population weights.

Here, we first demonstrate the feasibility of Bayesian inference based on large structural libraries from detailed all-atom simulations. By inferring ensembles from synthetic data we show that the method is capable of accurate recovery of population weights and ensemble sizes. We then investigate how noise in the experimental data impacts the accuracy of ensemble inference, showing that information encoded in energy functions can compensate for noisy SAS data. The inference machinery is then applied to evaluate conformational ensembles of two well-characterised proteins, previously studied by SAXS and NMR, each having two domains connected by a flexible linker: calmodulin (CaM) and a two-domain construct, designated ΔmC2, from the cardiac myosin binding protein C. A significant benefit of Bayesian methods is that multiple experimental observations along with simulations and force fields can be rigorously combined in both model selection and weight inference to gain insight into the underlying ensemble. This approach is exemplified in the study of our two example proteins where we demonstrate how data from SAXS, NMR and structural energy values of individual conformers can be combined into one probabilistic model for improved ensemble inference.

## Results

### Bayesian inference of conformational ensembles

We seek to determine optimal structural ensembles from experimental data by selecting conformers from a structural library and inferring their population weights. The experimental measurements generated by a discrete ensemble of conformers can be modelled as a weighted sum of measurements expected from each conformer

$$\vec{m}(x)\;=\;\sum_{i\;=\;1}^{n}w_{i}\vec{M}(x)$$ (1)

where $\vec{M}(x)$ is the expected measurement for a single conformer *i* over a sampling point x and *w*_*i*_ is the population weight of conformer *i*. For SAXS measurements $\vec{M}(x)\;=\;\vec{I}(q)$ where $\vec{I}(q)$ is intensity defined for scattering vector amplitude *q*. The objective of the Bayesian methodology is to infer the population weights *w*_i_ on the basis of experimental measurements $\vec{m}$ and a set of structural models, which can be done by employing Bayes’ theorem

$$f(\vec{w}|\vec{m},\text{S})\;=\;\frac{f(\vec{m}|\vec{w},\text{S})f(\vec{w}|\text{S})}{f(\vec{m}|\text{S})}$$ (2)

where $f(\vec{w}|S)$ is the prior probability of weights $\vec{w}\;=\;[w_{1},\;\ldots ,w_{n}]$, S = {*S*_1_, …, *S*_*n*_} is a structural library, $f(\vec{m}|\vec{w},S)$ is the likelihood of observing the measurements given the weights and set of structures, and $f(\vec{w}|\vec{m},S)$ is the posterior probability of the weights given the experimental measurements.

The likelihood function measures how well a given model matches experimental data. In our modeling, we assume that the experimental errors are normally distributed with standard deviations that can be estimated from the data, and that the individual data points are independent. We primarily focus on experimental data from SAXS but also employ chemical shift data from NMR. SAXS and NMR data can easily be combined by multiplying their respective likelihood functions.

Finally, we need to define a prior distribution over the weights $\vec{w}$. It is convenient to use Dirichlet distribution, which guarantees that weights sum up to 1

$$g(\vec{w}|\vec{\alpha },S)\;=\;\frac{\Gamma (\alpha_{0})}{\sum_{i\;=\;1}^{n}\Gamma (\alpha_{i})}\prod_{i\;=\;1}^{n}w_{i}^{\alpha_{i}-1}$$ (3)

where α_i_ are the parameters of the Dirchlet distribution and α_0_ is the sum of α_i_’s. At this stage we assume that all conformers are equally likely in the modeling and chose α_i_’s as the non-informative Jeffrey’s prior. However, if a more realistic energy function has been used to generate the structural library it is possible to bias the inference towards those conformers with favorable energies. In a scenario where several structurally different conformers have very similar scattering curves, such energy data can be used to select a more realistic ensemble. There are several different approaches that could be used to employ structural energy data in the ensemble inference. Our preference is to bias the prior probability distribution over weights by energy values from simulations. The structural energy values can be used to predict the population weights based on the Boltzmann distribution

$$w_{i}\;=\;\frac{e^{-(U_{ref}+U_{i})/kT}}{\sum_{i}^{n}e^{-(U_{ref}+U_{i})/kT}}\;=\;\frac{e^{-U_{i}/kT}}{\sum_{i}^{n}e^{-U_{i}/kT}}$$ (4)

where *U*_*i*_ is the energy of conformer *i*. *U*_*ref*_ can be thought of as a variable that shifts the energy measured by the energy function onto the absolute energy scale but does not affect the populations. By using a Dirichlet distribution with concentration parameters $\alpha_{i}\;=\;e^{-(U_{ref}+U_{i})/kT}$, the prior can be centered around the Boltzmann values, with *U*_*ref*_ controlling the sharpness of the distribution. We assign a uniform prior to the hyperparameter *U*_*ref*_ and treat it as sampling parameter.

Once likelihood and prior distributions are defined it is possible to evaluate the posterior probability distribution by employing Markov Chain Monte Carlo sampling. However, when large structural libraries are used there can be thousands of parameters in such probabilistic models, which make complete Bayesian inference computationally intractable. We therefore use variational Bayesian inference to shrink the size of the ensemble to a more tractable size range, at which point a complete Bayesian inference is applied to infer population weights.

### Model selection

The goal of model selection is to determine the size of the ensemble and which members of the structural library to include. In variational Bayes, the true posterior probability distribution is approximated by a distribution with a favorable mathematical form. The parameters of this approximate distribution are found by minimizing the difference to the true posterior. This can be achieved by minimizing the Kullback-Leibler (KL) divergence between the true and approximate distribution: two identical distributions have zero KL-divergence. The KL-divergence cannot be easily evaluated, but it turns out that minimizing the KL-divergence is equivalent to maximizing a lower bound on the value of the model evidence (ELBO, denominator in Eq 2):

$$f(\vec{m}|S)\;=\;\int f(\vec{m}|\vec{w},S)f(\vec{w}|\text{S})d\vec{w}$$ (5)

We can find an analytical form for ELBO, which means that the inference problem can be turned into an optimization problem that is much more computationally tractable than sampling.

Maximizing ELBO thus also leads to maximization of the model evidence function, which is a central property in Bayesian model selection. Consider two possible subsets of structures (or, mathematical “models”) S^(1)^ and S^(2)^ from a structural library. To compare the models, we can calculate the ratio of likelihoods of the competing models given experimental data (the Bayes factor)

$$\frac{f(S^{\left(1\right)}|\vec{m})}{f(S^{\left(2\right)}|\vec{m})}\;=\;\frac{f(\vec{m}|S^{\left(1\right)})}{f(\vec{m}|S^{\left(2\right)})}\frac{f(S^{\left(1\right)})}{f(S^{\left(2\right)})}\;=\;\frac{f(\vec{m}|S^{\left(1\right)})}{f(\vec{m}|S^{\left(2\right)})}$$ (6)

where the second identity comes from assuming that each model is equally probable *a priori*. Thus, finding the most likely model given the experimental data is identical to selecting the ensemble with the highest model evidence. As demonstrated by Fisher and colleagues [29], the variational approach can be used to build a straightforward model selection approach along these lines: with a given structural library the KL-divergence is minimized by maximizing the ELBO. Members of the ensemble with lowest population weights (below preset *w*_*cut*_ threshold) are pruned and the calculation is repeated on the reduced ensemble until the ELBO no longer increases, at which point the optimal ensemble has been identified.

To carry out the inference we need to approximate the posterior distribution over the weights $\vec{w}$. In the variational approach we assume that the posterior probability distributions over the weights can be well described by a Dirichlet distribution (Eq 3) and ELBO is maximized by optimizing the concentration parameters α_i_. The choice of the Dirichlet distribution to approximate the posterior results in a closed-form solution for ELBO [29]. Simulated annealing is then used to maximize with the respect to the concentration parameters α_i_. The population weights are then calculated as

$$w_{i}\;=\;\frac{\alpha_{i}}{\sum_{i}\alpha_{i}}$$ (7)

These weight estimates are compared to the cutoff value in the model selection algorithm.

### Model selection from synthetic scattering data

**Fig 1:**
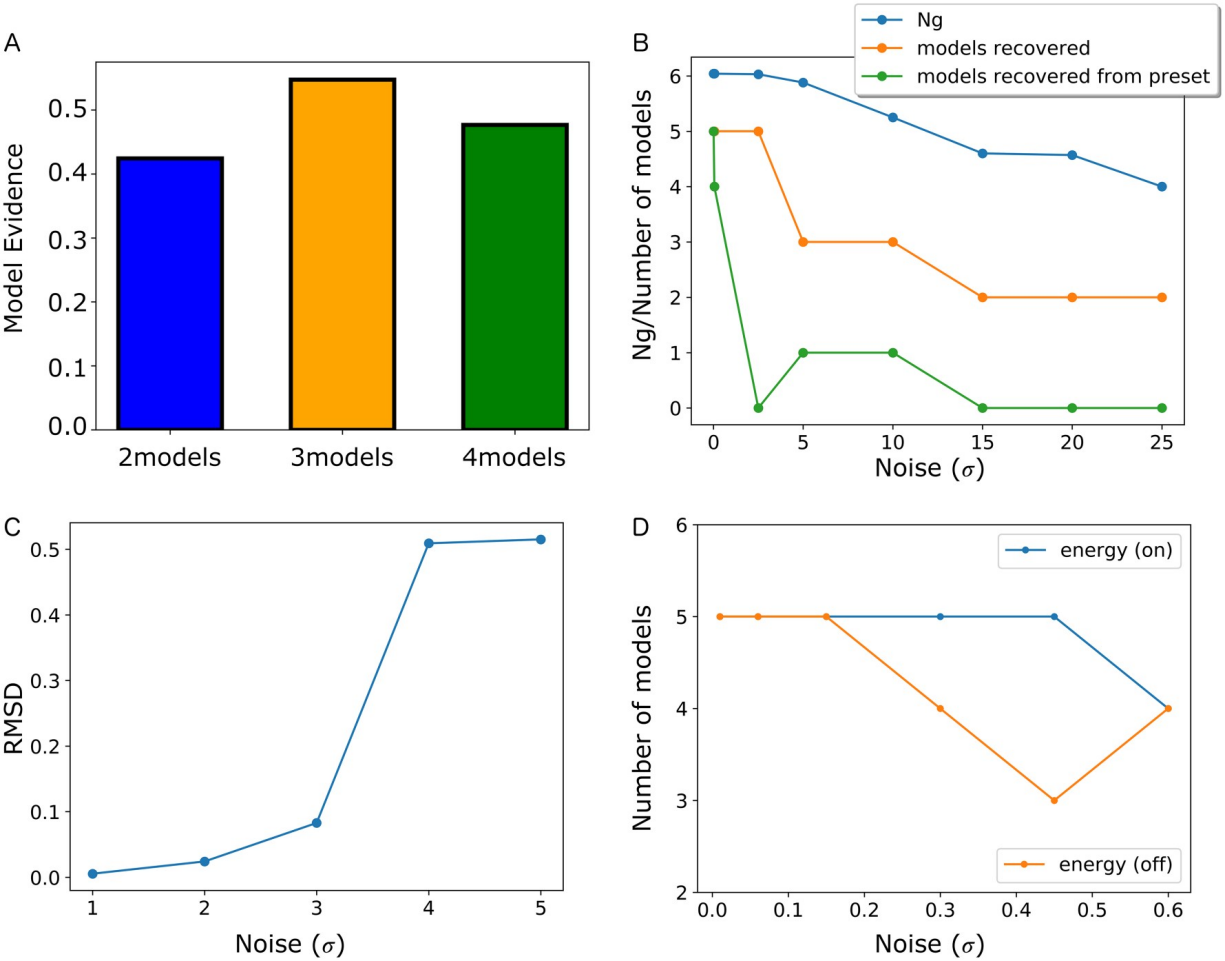
Benchmarking of VBI **(A)** Selection of optimal ensembles sizes using model evidence. Histogram shows maximum model evidence for all possible ensembles of 2–4 members for a synthetic ensemble consisting of 3 members selected from a library of 10 conformers. (**B)** Effect of noise on ensemble inference. The magnitude of noise is scaled in relation to the noise in ΔmC2 experimental data (σ = 1). Inferred number of models (orange), number of recovered models from synthetic ensemble and N_g_(blue) as function of simulated noise (σ). Synthetic ensemble was generated using 5 models with arbitrary weights and structural library consisted of 100 conformers. **(C)** Error in weights inference as function of noise. Root mean square deviation (rmsd) between simulated and inferred weights as function of simulated noise. Magnitude of noise defined as in **(A)**. (**D**) Inference with and without the Rosetta energies. Simulated ensemble with 5 members that are assigned equal population weights. Recovery of number of models in the synthetic ensembles from a library of 100 conformers in the presence (orange, conformers energies: -135.2, -140.0, -126.7, -125.5, -124.0) and absence (blue) of energy prior as a function of noise. Magnitude of noise defined as in **(B)**.

Our method enables optimal ensemble selection from large structural libraries using variational Bayesian inference. Before we demonstrate the full potential of the model selection, we first demonstrate the power of model evidence to identify optimal ensemble sizes when it can be accurately calculated (not approximated). To illustrate the concept, we generated synthetic data and a structural library of ten members from discrete structural models of the two-domain construct ΔmC2 from cardiac Myosin Binding Protein C (which will be described in more detail below in the context of the applications with real experimental data). We created an ensemble of 3 arbitrarily selected models from the set of ten and simulated a combined scattering curve for these models. Using these simulated data and a structural library of 10 members, we calculated the model evidence for all possible ensembles with 2,3 and 4 members. Fig 1A shows the maximal model evidence as a function of model size. As expected, model evidence picks out 3 as the most optimal ensemble size.

We then investigated the ability of VBI to accurately recover the correct ensemble and population weights using synthetic data based on a structural library of ΔmC2. From a larger structural library of 1000 conformers a smaller library of 100 was generated by selecting structures that covered a similar distribution of radius of gyration (*R*_*g*_) values to the larger library. From this subset a handful of structures (5 models) was selected, each with an arbitrarily chosen population, to generate synthetic experimental data. Gaussian statistical errors were added to the data according to the method described by Karaca et al. [30].

A key challenge in ensemble inference is to identify the optimal set of members. This step can be very difficult because even with a relatively small structural library of 100 members the number of possible ensembles is staggering; *e*.*g*. there are 10^10^ unique ensembles available having 1–7 members. S1 Fig illustrates the process of ensemble inference by the algorithm on synthetic data generated with 5 members and added synthetic noise starting from the 100-member structural library. VBI recovers the correct members of the ensemble and their corresponding weights. Although the recovery of weights in this example is impressive, there are a couple of caveats. One is that ensemble members with small population weights may be prematurely pruned during iterations of the ensemble selection algorithm. This simple algorithmic issue could be corrected by optimizing the threshold used to cull members from the ensemble. But there is also a more fundamental issue with uniqueness of the ensemble. In a bigger structural library, there will be conformers with nearly identical scattering profiles. As the size of the structural library increases, the exact identity of members in the ensemble may not be recovered. When we expand the library from 100 to 1000 members this behavior is indeed observed. However, the alternative ensembles recovered in this case have similar model evidence to the simulated ensemble and are thus equally optimal.

### Model selection from noisy data

Synthetic ensembles allow us to characterize the effects of experimental noise on the ensemble selection, such as reduced accuracy of population weights inference or a reduction in information content in the data that leads to a smaller number of members of the ensembles that can be supported by the data. Information content in a SAS curve has traditionally been estimated using information theory by calculating the number of Shannon channels needed to completely recover the data [31]. However, this approach does not take into consideration the effect of noise. Such effects can be evaluated by calculating the “number of good parameters”, *N*_*g*_, instead. *N*_*g*_ provides the number of parameters that can be determined from measurements and can be estimated from data using maximum entropy regularization [32]. Vestergaard and Hansen [33] have developed a Bayesian approach to evaluate *N*_*g*_ for SAXS data, an approach we employ here. Based on the synthetic ensemble with 5 members we increased the amount of synthetic noise applied to the data and calculated *N*_*g*_. VBI was then applied to these data to recover optimal ensembles. Fig 1B shows the size of the ensemble as a function of added noise. *N*_*g*_ for the simulated data is around 6 and drops down to 4 at the highest levels of noise. At lower noise levels all 5 ensemble members are recovered. However, increasing noise leads to smaller inferred ensembles with only two members at the highest noise levels. A second effect of increasing noise is a change in the identity of the recovered ensemble members. As the noise increases and the size of the ensemble is reduced, the original ensemble members are not necessarily part of the optimal ensembles.

To further investigate how noise affects the accuracy of inference we repeated the above model selection with synthetic data and signal-to-noise levels set with reference to the experimental data for ΔmC2 (described below). In Fig 1C the accuracy of the inferred weights, characterized by the root mean square deviation (rmsd) between simulated and inferred weights, is plotted as a function of increasing noise in the data. The results demonstrate that the inference is still very accurate up to three times the experimentally observed noise in our example ΔmC2. As the added noise increases beyond this value the number of inferred ensemble members decreases, which is the primary reason for the rapid increase in error in rmsd.

### Model selection with structural energies

So far, we have assumed that all conformers are equally likely in the modeling. However, we can also bias the inference with the energies generated for conformers from the structural library. In our simulations, *U*_*ref*_, which controls the strength of the prior, is selected by optimizing evidence using a variational Bayes approach. In this way, the uncertainty in the experimental data will automatically control the strength of the energy prior. This effect is demonstrated by carrying out inference with an energy prior that is centered around Boltzmann weights whose values differ from the simulated values. When the noise level is low and the information content high in the experimental data, the inference relies strongly on the experimental data with small rmsd differences between inferred and simulated weights. As the noise levels increase and the information content is reduced, the energy prior takes over and the weights move towards the values predicted by the Boltzmann distribution (S2 Fig).

By establishing the impact of inference with structural energies on the fixed set of models, we further investigate the power of using structural energies on model selection in the presence of experimental noise. In Fig 1D we show the result of the inference of a synthetic ensemble of 5 lowest energy conformers from a library of 100 members as a function of noise. In the absence of the energy prior, the number of recovered members from the simulated ensemble is reduced to 4 and 3 as the noise increases. With the energy prior turned on, the full ensemble is recovered at much higher levels of noise. This result is obtained even when the Boltzmann weights did not exactly match the simulated population weights. However, due to the different weights, the rmsd relative to the simulated weights is slightly higher with the energy prior turned on.

In order to demonstrate that the introduction of energy priors does not steer the resulting ensembles excessively towards the lowest energy structures, we added an energy refined conformer with substantially improved energy to the library. With this addition, there was little effect on the identity of recovered models (S3 Fig) and the trend observed in Fig 1D is retained.

### Weights inference using complete Bayesian inference

Once a smaller subset of models has been selected using VBI, we subject the optimal ensemble to Complete Bayesian Inference (CBI) to determine the population weights and their distributions. In general, a strong benefit of Bayesian inference is that we can go beyond single values (point estimates) for population weights and characterize the complete posterior probability distributions of inferred parameters. This step provides probability distributions over the individual weights in the ensemble, together with credibility intervals if requested.

It is also possible to characterize the uncertainty of the complete ensemble. Fisher and colleagues [29] developed a useful metric to measure the uncertainty of ensembles, the expectation value of the Jensen-Shannon divergence (JSD) relative to the optimal weights over the posterior distribution

$$\sigma_{{\vec{w}}_{B,S}}\;=\;\int JSD(\vec{w,}\;{\vec{w}}_{B,S})f(\vec{w}|\vec{m,}S)d\vec{w}$$ (8)

where $JSD(\vec{w},{\vec{w}}_{B,S})\;=\;\frac{1}{2}\sum_{i\;=\;1}^{n}{\vec{w}}_{i}{log}_{2}(\frac{{2\vec{w}}_{i}}{{\vec{w}}_{i}+{{\vec{w}}_{B,S}}_{i}})+\frac{1}{2}\sum_{i\;=\;1}^{n}{{\vec{w}}_{B,S}}_{i}{log}_{2}(\frac{{{2\vec{w}}_{B,S}}_{i}}{{\vec{w}}_{i}+{{\vec{w}}_{B,S}}_{i}})\;$ and ranges between 0 and 1 for two maximally identical and different vectors, respectively, which means that also $\sigma_{{\vec{w}}_{B,S}}$ falls within this range.

We carry out the complete Bayesian inference using the No-U-Turn sampler (NUTS) [34] implemented in the Stan software library [35]. NUTS is an extension of Hamiltonian Monte Carlo, an MCMC algorithm that avoids the random walk behavior and sensitivity to correlated parameters that often plague MCMC inference.

To validate the inferred ensembles, it is useful to carry out posterior predictive checks [36]. This check can be achieved by repeatedly simulating scattering curves with the inferred ensemble model and then comparing these to the experimental data. As seen in S4 Fig, experimental curves simulated by our statistical model closely match experimental data. For example, when ensembles are inferred using an unsuitable error model, it is immediately obvious in these predictive checks.

### Application of Bayesian inference method to experimental data

Having characterized the performance of Bayesian inference methods on synthetic data sets with relatively small structural libraries, we now apply the method to two experimental systems from our previous work: a two-domain protein calmodulin (CaM) [14], and the two-domain construct, ΔmC2, from the cardiac myosin binding protein C (cMyBP-C) [37].

### Calmodulin

CaM is the major intracellular Ca^2+^ receptor that binds to a diverse array of target proteins (numbering in the 100s) to regulate their activities in response to Ca^2+^ signals (reviewed by Tidow et al. and Crivici et al. [10, 38, 39]). The crystal structure of CaM [40] shows a mostly α-helical structure with an unusual dumbbell shape formed by two globular, cup-shaped domains connected by an extended α-helix of 7–8 turns. Upon Ca^2+^-binding at the base of each cup-shaped domain a hydrophobic cleft, which is essential for target binding, opens via the concerted movements of pairs of helices. NMR studies showed the interconnecting helix is broken in solution by a short sequence of four highly mobile amino acids [41] that allow CaM to orient and position the hydrophobic clefts and additional contact regions to accommodate structurally diverse targets. Thus CaM’s structure encodes for both structural diversity and specificity for target binding. CaM was chosen as a test case because it is an extensively characterized protein and understanding the nature of the conformations present in solution for uncomplexed CaM and how that conformational equilibrium is influenced by the presence of binding partners is thus of considerable interest. It is also a popular target for molecular dynamics (MD) simulation, including studies aimed both to gain insight into CaM dynamics (*e*.*g*. [42–47] and to test MD results against experiment (*e*.*g*. [48]).

To generate a library of structurally and energetically reasonable conformers of CaM (which herein refers to the Ca^2+^-saturated form with the four Ca^2+^ sites fully occupied, and thus primed for target binding) we developed a Monte Carlo based simulation of linker flexibility. A sampling protocol was developed in the Rosetta macromolecular modeling package where the torsion angles in the linker segment were sampled in a Monte Carlo simulation followed by an all atom energy refinement of the linker segment and the neighboring residues. In addition, the 3 N-terminal residues and the last C-terminal residue (lysine 148) missing in the crystal were modelled *de novo* as well. Around 10000 models were generated by this procedure and a structural library was created by taking the lowest energy 1000. The distribution of *R*_*g*_-values in the structural library for all 10000 models and after applying energy filter is shown in S5A and S5B Fig. The *R*_*g*_ distribution for the lowest energy subset models covers the same *R*_*g*_ range as for the complete library but is slightly more peaked.

Using a high quality SAXS data set of CaM obtained using in-line SEC (size exclusion chromatography) at the Australian Synchrotron [14] and NMR chemical shift data [49], we performed model selection using VBI with the 1000 lowest energy conformers. We evaluated four inference scenarios using: 1) SAXS data only, 2) SAXS data + Rosetta energies, 3) SAXS data + chemical shifts and 4) SAXS data + chemical shifts + Rosetta energies. Once VBI converged and the ensemble consisting of a few members was selected, we used CBI to infer population weights and their distributions. While condensing the probability distributions into point estimates (single values) of parameters is undesirable in general, it is sometimes convenient in comparison with alternative methods to easily summarize error residual plots and evaluate other figures of merit. For this purpose, we calculate scattering curves for inferred ensembles using point estimates of parameter taken from the VBI inference. These point estimates are found as the parameters (e.g. population weights) that maximizes the ELBO metric.

**Fig 2:**
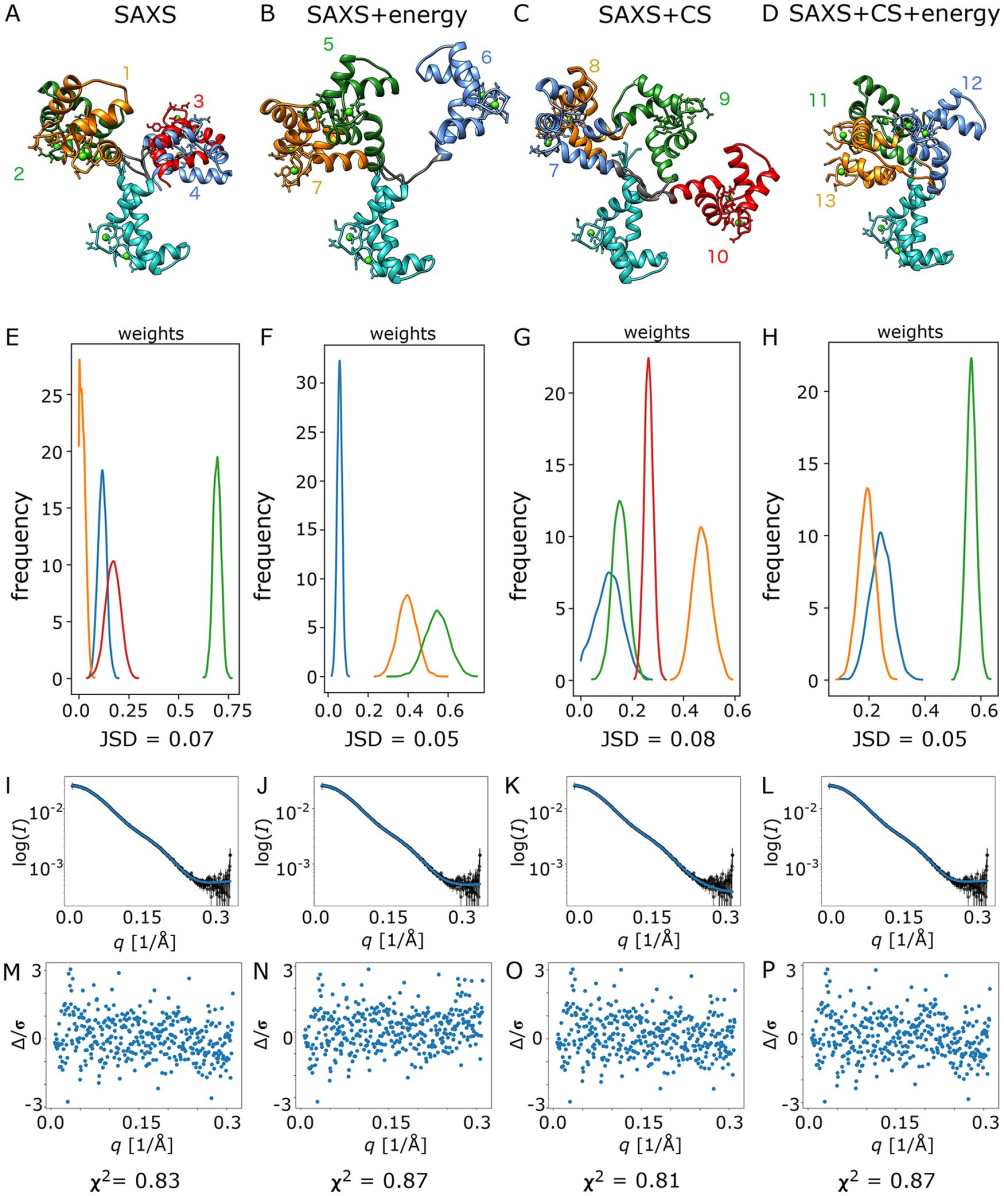
Bayesian inference of CaM conformational ensembles from (A) SAXS data only, (B) SAXS data with Rosetta energies, (C) SAXS and chemical shift data only and (D) SAXS and chemical shift data with Rosetta energies. (E-H) Population weight distributions for inferred ensembles from all four inference scenarios. (I-L) Ensemble model fit to SAXS data from the point estimate of population weights from VBI. (M-P) Error weighted intensity difference plots for each ensemble model fit to the SAXS data. Structural models were aligned on N-terminal domain (cyan). Different C-terminal orientations (various colors and numbers 1–13) correspond to different conformers.

Each of the ensembles inferred with the prior distribution unbiased by the inclusion of energies (scenarios 1 and 3) consists of 4 members (Fig 2A and 2C), while the scenarios with the Rosetta energies included for the prior distribution (2 and 4) result in 3 members (Fig 2B and 2D). The drop in the number of members upon inclusion of energy priors is due to the peaked energy landscape, which reduces the number of possible solutions and also results in faster convergence of selection algorithm (S1 Table). Inferred weights for each scenario have relatively peaked distributions (Fig 2E–2H) and JSD ranges from 0.05 to 0.08, which means that there is high certainty in the predicted parameters given the ensemble of models and the experimental errors.

The predicted scattering profile from each of the ensembles for the different inference scenarios matches the SAXS data well, as illustrated in Fig 2 (panels I-L) and a number of statistical measures. The reduced χ^2^ value obtained for the predicted scattering profile for each ensemble is in the expected range for an excellent model fit to the data (*i*.*e*. near 1; in this instance in the range 0.81–0.87). The use of energy priors leads to a small increase in χ^2^ in the presence and absence of the CS data. The addition of CS data slightly improves the fit to the SAXS data compared to when SAXS data is used alone, indicating the data sources are at least not in conflict and potentially may be reinforce each other. The absolute value of χ^2^ depends critically on accurate counting statistics and error propagation. Further as a global parameter, χ^2^ will not identify significant regions in *q*-space of mis-fit. The predicted scattering profiles were therefore also assessed (1) using an error weighted difference plot over the measured *q*-range and (2) with the recently developed correlation map (CorMap) test [50] that is independent of the errors and identifies regions of misfit with a significance test. Simply put, CorMap identifies the longest stretch of data points that lie on one side of the model profile and provides a probability (*P*) for that occurrence given the number of points in the data set. Consistent with the observed flatness of the error weighted model versus experiment intensity difference plots (Fig 2M–2P) over the entire *q*-range, CorMap gives *P*-values indicating high confidence in the model fit (0.53–0.96). Thus by all measures each of the inferred ensembles are in excellent agreement with the SAXS data, have high certainty in the predicted parameters. Arguably, one could conclude that the “best-fit” to the SAXS data is obtained for scenario 3 (SAXS data + CS) as assessed by the lowest χ^2^ value combined with the highest *P*-value and the fact that the longest stretch of points on one side of the model profile lies, uniquely among the four scenarios, in the high-*q* background scattering region. All parameters for the inferred ensembles are summarized in S2 Table.

Examining the CaM conformers in each selected ensemble, with a single exception, the *R*_*g*_ values are all in the relatively narrow range 20.6–23.0 Å (S2 Table). This range is consistent with the original SAXS study of CaM in solution [51] that concluded that the CaM lobes are on “average” reoriented and closer together in solution compared to the crystal structure (PDB 1CLL) with its fully extended helical inter-domain connector (*R*_*g*_ = 22.7 Å). The main distinction among the inferred *R*_*g*_ distributions is that the inclusion of Rosetta energies results in a higher proportion of more compact structures within this range, although the SAXS + Rosetta energies inference also yields the most extended conformer with an *R*_*g*_ value 26.0 Å, albeit with a relatively low population weight (0.06 ± 0.1).

The conformers of the inferred CaM ensembles all show variable orientations of the N- and C-terminal target-binding hydrophobic clefts and variable degrees of extension in the flexible linker (Fig 2A–2D). Inspection of known crystal or NMR solution structures of CaM complexed with target binding proteins or domains also reveals conformers with highly variable domain dispositions (reviewed in Tidow et al. [10]). They also include CaM conformers that are significantly more compact or more extended than either the crystal structure or those present in the majority conformers from inferred ensembles; *e*.*g*. CaM with its binding domain in myosin light chain kinase has an *R*_*g*_ of 17 Å with its two globular lobes wrapped tightly around the helical binding domain (PDB 2LV6) while the 20 lowest energy NMR structures for CaM complexed with its binding domain from Munc13 (PDB 2KDU) includes CaM conformers with *R*_*g*_ values as large as 26.4 Å. A systematic comparison of all CaM conformers represented in complexes with binding partners in the PDB identified 1 crystal structure (4DJC) and 3 NMR solution structures (1CFF, 2KDU and 1L53) with similar dispositions of the CaM domains as assessed by rmsd values for Cα coordinates in the range 4.6–7.3 Å (S3 Table). Of this set of structures, only the 2KDU structure has both CaM binding domains involved in the target domain interaction, the remaining three only involve C-terminal domain binding, and the 1CFF crystal structure has the fully extended helical inter-domain connector, similar to the Ca^2+^-CaM 1CLL structure. A library of CaM structures was generated from all the structures in the PDB of CaM complexed with a target involving interactions with both of CaM’s N-and C-terminal domains. When inference is carried out with this structural library, the resulting ensemble cannot describe the experimental data well.

In sum, each of the inferred ensemble models show variable dispositions of the target-binding hydrophobic clefts and includes some conformers that have similar dispositions to conformers observed in crystal or NMR solution structures of CaM complexes. Further, the *R*_*g*_ values for the ensemble model conformers are all in a range that is within the range observed in these structures. However, each inference scenario results in distinct set of conformers in an ensemble that fits the available data more-or-less equally well. Thus, while the model evidence justifies an ensemble model of 3–4 models, the solution is not uniquely defined by the available experimental data.

This ambiguity can be potentially removed by introducing additional experimental data that informs on inter-domain orientation. Such information is found in data from NMR Paramagnetic Contact Shifts (PCS) and Residual Dipolar Couplings (RDCs) measurements for example, and has proven to be useful in combination with SAXS [52, 53]. Developing methods required to incorporate this type of data into our statistical framework is beyond the scope of this study. However, we can test how well the ensembles inferred in this study explain experimental PCS values from paramagnetic data. We compared predicted values from inferred ensembles with available paramagnetic data for Tb (terbium(III)), Dy (dysprosium(III)) and Tm (thulium(III)) bound to the N-terminal domain of CaM derivatives [54]. The predicted ensembles do not fit particularly well with the PCS data for the C-terminal domain. This could be because PCS reports on orientational information not available in SAXS and chemical shift data. However, the conditions at which the PCS data is significantly different than used for SAXS (pH (6.5 vs 7.5) and ionic strength (300 vs 400 mM)). Since CaM is very negatively charged [55], it cannot be ruled out that the ensembles are different at these two conditions.

It is the hydrophobic cleft in the C-terminal lobe of CaM that is generally the initial recognition site for target binding in a two-step binding process whereby subsequent N-terminal lobe binding is necessary for full cooperative target binding. Further, it is not unusual for the CaM binding sequences to be anchored via other interactions within the target proteins; *e*.*g*. in myosin light chain kinase the CaM-binding domain has to be released and translocated away from the kinase’s catalytic cleft [56], and in CaM’s interaction with the MA protein from HIV-1 the two-tryptophan’s that bind to the C- and N-terminal domains of CaM are deeply buried in the helical head domain of MA [57, 58]. The ensemble models thus support the idea that the flexible linker in CaM primarily allows the hydrophobic clefts to reorient independently. This mobility enables target recognition and binding by the C-terminal hydrophobic cleft of CaM that in turn triggers the unfolding and folding events required to form the interaction surfaces. Such a process is consistent with the conclusions of Liu and colleagues from their molecular dynamics study of CaM binding to its binding domain in skeletal muscle myosin light chain kinase, that the binding process is “quite complex with the mixture of induced fit, conformational selection, and simultaneous binding–folding.” [42].

### *ΔmC2* from the cardiac myosin binding protein C

Our second example of the application of VBI to experimental data considers ΔmC2 from cMyBP-C, which has never been crystallized but our NMR solution structure (PDB:2KDU) [37] reveals it to have a two-domain structure with a 7-residue flexible linker. The cMyBP-C is a modular protein with eleven predominantly β-structured immunoglobulin (Ig) or fibronectin (Fn) domains (designated C0 through C10) and a 100-amino acid sequence between C1 and C2 that contains cardiac specific phosphorylation sites and is mostly unstructured (referred to as the “motif” or m-domain) [59, 60]. Found in the cross-bridge bearing C zone of the A band of the muscle sarcomere, cMyBP-C interacts with both thick and thin filaments and has both structural and regulatory functions [61]. It exercises its regulatory function via alternate myosin/actin interactions with its N-terminal domains (C0-C1-m-C2), with phosphorylation of the motif implicated in the switching [62–64]. The ΔmC2 construct includes the loosely structured C-terminal region of the m-domain that is a tri-helix bundle [65] with a tightly structured C2 that has an Ig-type fold [66]. Our NMR structure showed the same folded tri-helix bundle as previously determined by NMR and the C2 domain connected by a 7-amino acid linker that is highly mobile, and yet there is a surprisingly high degree of sequence conservation in this linker sequence across all known chordates [37]. Further, the linker includes sites of severe disease-linked mutations and also forms part of the interface of a stable, Ca^2+^-dependent interaction with CaM. These observations, combined with evidence implicating ΔmC2 in actin binding, led us to postulate that, like CaM, the flexible linker region of ΔmC2 may facilitate its role as a polymorphic binding domain that interacts with multiple proteins to regulate muscle action in the sarcomere [37].

SAXS and NMR chemical shift data for highly purified ΔmC2 were from [37]. The SAXS data were of good quality, also from the Australian Synchrotron, but measured in a typical batch mode without the benefit of in-line SEC. A small concentration dependence was observed in the lowest-*q* data that, while corrected by a linear extrapolation to zero concentration, amplified the errors in this region.

**Fig 3:**
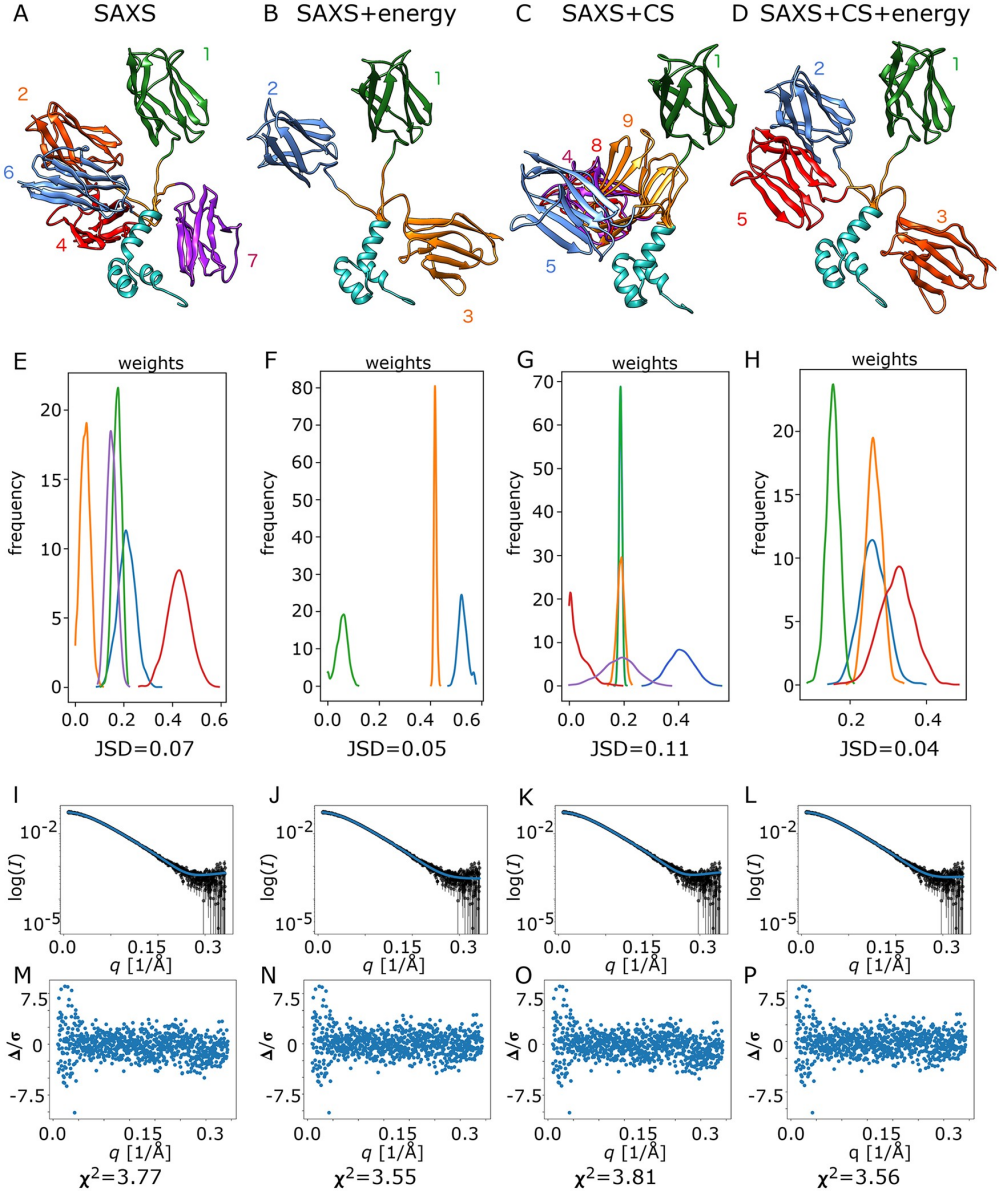
Bayesian inference of ΔmC2 conformational ensemble from (A) SAXS data only, (B) SAXS data + Rosetta energies, (C) SAXS data + CS and d) SAXS data + CS + Rosetta energies. (E-H) Population weight distributions for inferred ensembles in all four inference scenarios in A. (I-L) Ensemble model fits to SAXS data based on the point estimate of population weights from VBI. (M-P) Error weighted intensity difference plots for each ensemble. Structural models were aligned on N-terminal domain (cyan). Different C-terminal orientations (various colors and numbers 1–9) correspond to different conformers.

Following the procedure described for CaM, and assuming two stable folded domains connected by a 7-residue linker, we generated a structural library of 1000 lowest energy conformers using the Rosetta protocol and ran the same 4 inference scenarios: 1) SAXS data only, 2) SAXS data + Rosetta energies, 3) SAXS data + chemical shifts and 4) SAXS data + chemical shifts + Rosetta energies. The ensembles inferred in scenarios 1 and 3 consist of 5 members (Fig 3A and 3C), while scenarios 2 and 4 (Fig 3B and 3D) yield 3 and 4 members, respectively. Similar to CaM, model selection when Rosetta energies are included in the prior leads to a smaller subset of inferred models.

The *R*_*g*_ range in each of the inferred ensembles is similar (~17–27 Å). As was observed for CaM, inclusion of Rosetta energies distributions significantly alters the weighting of more compact structures to more extended ones (0.80–0.84 and 0.42–0.58 without and with energy priors, respectively). In contrast to the CaM, however, the change in weights with energy priors shifts the distribution to an increase in the proportion of more extended structures. The most highly extended conformer (*R*_*g*_ = 27.0 Å) appears in all four variants (model 1 (green) in Fig 3A–3D) though its population weight with the inclusion of both CS and energy priors (inference 2) is significantly smaller than in the other ensembles. In all scenarios except 3, which is the only one for which a conformer with the intermediate *R*_*g*_ value (24 Å) is absent, inferred weights have a peaked distribution over the weights and JSD ranges from 0.04 to 0.07. The JSD is slightly higher for variant 3 (0.11), primarily due to the long tail of the lowest weight, even so it still corresponds to an ensemble that is well-defined.

Similar to CaM we can assess the fit to data based on a point estimate of weights from VBI (Fig 3I–3L). Compared to CaM χ^2^ are considerably higher (ranges from 3.55 to 3.81), although error weighted difference plots (Fig 3M–3P) and CorMap *P*-values values (0.19–0.81) indicate good fits to the data over the measured *q*-range with no statistically significant specific region of mis-fit. We can thus conclude that the errors propagated from counting statistics were on this occasion underestimated, which has been a common issue for SAS data. χ^2^ drops when simulations include Rosetta energies in the prior over weights (variant 2 and 4). In interpreting this result, it is important to highlight that the ensemble and population weights are not selected by minimizing χ^2^. The drop in χ^2^ is the result in improved quality of the ensemble and highlights how multiple data sources can work together to provide a better-defined ensemble. Inference with chemical shift data leads to slightly increased χ^2^ for SAXS of 3.81, suggesting that the ensemble observed by SAXS and NMR chemical shift may differ somewhat, potentially due to subtly different solution conditions. The detailed values of inferred parameters can be found in S2 Table. To further investigate this issue, we ran CBI with the ensemble only selected from SAXS data with the four different data scenarios as presented above (results found in S4 Table, which also presents values for CaM). With the SAXS ensemble, inference of SAXS+NMR data is essentially identical to when only SAXS data is used. However, no improvements in the inference is observed when the Rosetta energy is used in this scenario. This highlights that differences with or without NMR and Rosetta energies is a consequence of identifying different conformers from the structural library with the additional data.

The ensemble members in each of the scenarios 1, 2 and 4 adopt 3 distinct conformations that upon aligning the tri-helix bundle form an approximate cross-like configuration, while those from scenario 3 form an approximate T-shaped configuration (Fig 3A–3D). However, given that the inference with energy priors have better match to SAXS data as well as lower JSD values we can conclude that the ensemble with cross-like conformation is more likely. Much less is known about ΔmC2 and its putative binding partners. The measured binding affinities are moderate (~100 nM) compared to CaM (~nM) [37] and, to date, there is no evidence for a common recognition motif. The ensemble modelling indicates that the longer flexible linker in ΔmC2 compared to CaM allows for significantly greater flexibility and relative positioning of its two domains, and more highly extended conformers are favored. Such an ensemble may be optimized for binding targets with moderate affinity where there is not a common initial recognition motif, and the binding process will also involve a mixture of induced fit, conformational selection, and simultaneous binding–folding.

### Comparison with other methods for inferring conformational ensembles

Many methods have been proposed for building conformation ensembles from SAS data. Typically, ensembles have been optimized by minimizing χ^2^. The fits are then characterized by visualization of fitting residuals. We compared the results from point estimates of weights from VBI with two popular methods for conformational ensembles modeling from SAS data: Ensemble Optimization Method or EOM [67] and MultiFoXS [21]. The results were summarized in terms of *R*_*g*_ distributions, number of ensemble members, χ^2^ and CorMap *P*-values (S5 Table). Focusing on the CaM ensembles obtained with SAXS-only data, with and without energies for the VBI ensembles, we see a striking similarity between the *R*_*g*_-values of conformers and weights between MultiFoXS and VBI for SAXS-only results. In contrast, the EOM and SAXS+Rosetta energies ensembles are more similar to each other, differing from the MultiFoXS results in the relative proportions of the more compact and more extended conformers. The inclusion of CS data does not significantly alter the VBI results in terms of *R*_*g*_ values and weights. For MultiFoXS, the minimal number of conformers required to minimize χ^2^ is selected and all structures that have correct stereochemistry, while for EOM a genetic algorithm is used to find an ensemble that minimizes χ^2^ and flexible regions are treated simply as a self-avoiding polyglycine chain. Thus, as might be expected, the number of ensemble members selected by VBI is much smaller than the number of representative structures selected by EOM but larger than for MultiFoXS. In the case of EOM the *R*_*g*_ distribution for the ensemble is a continuous double-peaked distribution that is represented by 13 conformers from this distribution, which is more than twice the number from the other methods.

While we have compared the χ^2^ values for the ensemble model fits to the SAXS data here, it is important to keep in mind that in contrast to EOM and MultiFoXS, the Bayesian approach does not select ensembles and weights based on direct minimization of χ^2^/χ and uses chemical shift and energy data in addition to data from SAXS in the inference. Nonetheless, by this comparison we see that the resulting χ^2^ values for the SAXS data fits are similar those obtained using EOM and MultiFoXS.

## Discussion

Small angle scattering data can provide structural insights into conformationally heterogeneous biological samples. Due to its inherently low information content, SAS data typically must be complemented with structural modeling to draw biologically relevant conclusions. While we want to extract as much information from the data as possible, care must also be taken to avoid overfitting. In ensemble inference there are two areas where overfitting may become a problem. First, with structural libraries containing thousands of members the number of modeling degrees of freedom significantly exceeds the information content in the data and this can result in inferences of overly complex ensembles. Second, by optimizing model parameters directly with respect to χ^2^ there is a risk of fitting to noise rather than signal in the experimental data.

Model evidence provides a principled approach to balance model complexity with fit to experimental data. We demonstrate that the approach can identify the optimal number of members using simulated ensembles with a known ensemble size. Model evidence also enables investigation of how experimental noise affects the inference of optimal ensembles. Our results show that although the ensemble inference is robust to high levels of noise, increasing noise eventually leads to the reduction of the information content in the data and smaller ensembles sizes that can be supported by data. Encouragingly, the analysis of the experimental data sets reports optimal ensemble sizes that are similar to the values obtained from the analysis of the number of good parameters (*N*_*g*_) suggesting that a good balance between model complexity and fit to data is reached. Model evidence is only one of several approaches for model selection employed in Bayesian inference. We have also employed model selection using WAIC and PSIS-LOO [68] but found that they did not result in stable ensemble inference.

In the simulation experiments with synthetic data, the exact identity of members in the optimal ensemble could be inferred from SAS data alone, except when the added noise became high. However, in scenarios with experimental data and large structural ensembles we do not necessarily expect there to be single optimal solution and many competing ensembles may equally well describe the experimental data. This result is not surprising as many different conformations can give rise to the same scattering profile. This is a fundamental consequence of the three-dimensional averaging of coordinates in SAS and not something that can be tackled with improved inference methods.

Bayesian approaches have some inherent properties that provide protection against overfitting to noise by balancing the fit to experimental data with information encoded in prior distributions over model parameters. The protection from the prior is particularly important in situations where the amount of experimental data is limited. Another benefit of the Bayesian methodology is that it returns probability distributions over modeling parameters rather than point estimates. Point estimates of population weights are a convenient approach to summarize results but represents an unnecessary reduction of information. The posterior probability distributions provide information about uncertainty of individual population weights. This can be complemented by the JSD metric that summarize uncertainty over the complete ensemble. We find small JSD values overall, suggesting relatively well-defined ensembles. Altogether, the posterior probability distributions and the JSD metric gives a full picture of the uncertainties in the ensemble inference given the available data.

Our approach for ensemble inference involves two separate stages. First, fast model selection is carried out using a variational approach that enables Bayesian inference with structural libraries consisting of thousands of members. This is followed by a complete inference the selected set using a full Bayesian inference. Comparison of the weight inference for CaM and ΔmC2 using the variational and complete suggests that the two approaches gives highly similar results, indicating that the approximations used in the variational method do not lead to any significant inaccuracies.

A powerful approach to better define ensembles is to include additional data into the inference and thereby increasing the information content. An additional benefit is that different data sources can provide different types of structural information. SAS provides information about relative positions of atoms in a structure. NMR chemical shift data on the other hand provides information about local structure of the protein while energies calculated through a force field or energy function provides information about stereochemistry and intermolecular interactions in the protein. The Bayesian approach straightforwardly enables the use of several information sources simultaneously in the inference. Our study of the two-domain proteins CaM and ΔmC2 with data from SAXS and NMR chemical shifts as well as Rosetta structural energies shows that for ΔmC2 that had higher levels of noise in the low-*q* SAXS regime, the use of Rosetta energy information leads to a significant improvement of the inference. The resulting ensembles have more peaked population weights distributions, better fit to the SAXS data (measured through χ^2^), fewer members and the Monte Carlo simulations converge faster. For the more ideal CaM data, we also observe more peaked probability distributions, fewer member and faster simulation convergence but see no improvement with the inclusion of energy priors in model fit to SAXS data measured through χ^2^. The inferred ensembles using SAXS only, SAXS+chemical shifts and SAXS+chemical shifts+structural energy have some conformers in common, but are different enough to present an alternative view of the conformational states of the proteins. Because the different inference scenarios are based on different data input, it is not straightforward to compare them statistically. Nonetheless, the ensemble inferred from the SAXS+chemical shifts+structural energies has the strong benefit that the conformers are consistent with the distance distributions measured through SAXS, the torsional preferences of the linker assessed by NMR and are energetically and stereochemically realistic through the use of the Rosetta energy values. When SAXS data is used alone, there are many ensembles with almost identical model evidence. Because of the lack of orientational information in the SAXS data, such ensemble can be quite different. The additional information from NMR and Rosetta can then tip the balance between these competing ensembles.

In reality we do not expect proteins with flexible linkers to populate only a discrete number of conformational states. The inferred ensembles represent a simplified model for explaining the dominant conformational states adopted by the protein. The small ensemble sizes are a reflection of the limited information content in the data which is not sufficient to infer more detailed picture of the conformational landscape. A fuller picture of the conformational ensemble could emerge if discrete structural library is replaced by a continuous model for structure. Antonov et al. have developed a probabilistic model for protein structure that enables sampling of conformations of the protein during ensemble inference [25], a method that does not rely on structural libraries. The challenge in employing such approaches is the development of probabilistic models over structure that samples energetically realistic protein conformations. For this reason, the use of structural libraries generated by atomistic force fields and energy functions still represent a useful strategy for inference of structural ensembles. Further research is necessary to develop approaches that combines the rigor of complete Bayesian inference with the structural and energetic realism encoded in force fields and energy functions.

## Methods

### Bayesian inference

In order to apply Bayes’ theorem (Eq 2) to infer the population weights *w*_i_ on the basis of experimental measurements $\vec{m}$ and a set of structural models *S*, we need to state the prior probability $f(\vec{w}|S)$, and the likelihood function $f(\vec{m}|\vec{w},\text{S})$. We define a prior probability over the weights $\vec{w}$ as Dirichlet distribution (Eq 3). The α_i_ parameter that defines Dirichlet distribution is either chosen to assume that all conformers are equally probable (non-informative Jeffrey’s prior) or to bias toward lower energy conformations from Rosetta simulations. For the non-informative prior the probability density function is defined as:

$$f(\vec{\text{w}}|\text{S})\;=\;\frac{\Gamma (n/2)}{n\Gamma (1/2)}\prod_{i\;=\;1}^{n}w_{i}^{-1/2}$$ (9)

However, when Rosetta energies are used the prior probability equals to:

$$g(\vec{\text{w}}|\text{S})\;=\;\frac{\Gamma (\beta_{0})}{\sum_{i\;=\;1}^{n}\Gamma (\beta_{i})}\prod_{i\;=\;1}^{n}w_{i}^{\beta_{i}-1}$$ (10)

where $\beta_{i}\;=\;e^{-(U_{ref}+U_{i})/k_{B}T}$, *U*_*ref*_ is the Boltzmann reference energy, *k*_*B*_ Boltzmann constant and $\beta_{0}\;=\;\sum_{i\;=\;1}^{n}\beta_{i}$.

The likelihood function describes uncertainty in experimental data. For SAXS data with normally distributed errors it is defined for each measurement *m*_*j*_ as a Gaussian density function:

$$f(m_{j}|\vec{w},\lambda )\;=\;\frac{1}{\sqrt{2\pi \varepsilon_{SAXS}^{2}}}exp\;(-\frac{(m_{j}-\lambda \sum_{i}^{n}w_{i}I_{ij}{)}^{2}}{2\varepsilon_{SAXS}^{2}})$$ (11)

where *λ* is a scaling factor, *I*_*ij*_ is a SAXS intensity calculated from the ensemble and *ε*_*SAXS*_ is the experimental error. We assume that measurements are independent and the joint likelihood is the product of individual likelihood functions:

$$f^{SAXS}(\vec{m}|\vec{w})\;=\;\prod_{j\;=\;1}^{N}f(m_{j}\vec{w},\lambda )$$ (12)

where *N* is the number of experimental measurements.

The Bayesian framework provides an easy approach to add structural information from different experimental sources. In the case of NMR chemical shifts measurements, we also assume that measurements are normally distributed and uncertainty of theoretical prediction of chemical shifts *ε*_*CS*_ can be summed up with experimental errors *ε*_*pre*_.

$$f_{NMR}(m_{j}|\vec{w})\;=\;\frac{1}{\sqrt{2\pi }\sqrt{\varepsilon_{CS}^{2}+\varepsilon_{pre}^{2}}}exp\;(-\frac{(m_{j}-\sum_{i}^{n}w_{i}C_{ij}{)}^{2}}{2{(\varepsilon }_{CS}^{2}+\varepsilon_{pre}^{2})})$$ (13)

where *C*_*ij*_ are chemical shifts calculated from the ensemble. Similar to SAXS data we assume that NMR chemical shift measurements are independent and joint probability $f^{NMR}(\vec{m}|\vec{w})\;$ is the product of individual likelihood functions.

$$f(\vec{m}|\vec{w})\;=\;f^{SAXS}(\vec{m}|\vec{w})∙f^{NMR}(\vec{m}|\vec{w})$$ (14)

### Variational Bayesian inference

The overall goal of variational Bayesian inference is to maximize the model evidence $f(\vec{m}|S)$. This is typically intractable, but we can find a lower bound for model evidence (ELBO) by introducing an approximate posterior $g(\vec{w}|\vec{\alpha },S)$ and applying Jensen’s inequality to the model evidence and maximize that instead [29]:

$$\log\;f(\vec{m}|\text{S})\;=\;\log\;\int g(\vec{w}|\vec{\alpha },\text{S})\frac{f(\vec{m}|\vec{w},\text{S})f(\vec{w}|\text{S})}{g(\vec{w}|\vec{\alpha },\text{S})}d\vec{w}\geq \int g(\vec{w}|\vec{\alpha },\text{S})\log\;\frac{f(\vec{m}|\vec{w},\text{S})f(\vec{w}|\text{S})d\vec{w}}{g(\vec{w}|\vec{\alpha },\text{S})}d\vec{w}\;\equiv \;-L(\vec{\alpha }|\text{S})$$ (15)

ELBO is determined by maximization of $-L(\vec{\alpha }|S)$ or minimization of $L(\vec{\alpha }|S)$ (Eq 16) through the choice of the parameters of the approximate distribution $g(\vec{w}|\vec{\alpha },S)$. In this way the parameters of $g(\vec{w}|\vec{\alpha },S)$ are chosen to minimize the KL divergence to the true posterior $f(\vec{w}|\vec{\alpha },S)$. The choice of $g(\vec{w}|\vec{\alpha },S)$ as a Dirichlet distribution enables a closed form solution for $L(\vec{\alpha }|S)$. The derivation for NMR chemical shift data can be found in Fisher et al. [29]. We modified the method to accommodate SAXS data:

$$\begin{array}{r} L\left(\alpha ,S\right)=\log\frac{\Gamma \left(\alpha_{0}\right)}{\Gamma \left(\frac{n}{2}\right)}+\overset{n}{\underset{i=1}{\sum }}\log\frac{\Gamma \left(\frac{1}{2}\right)}{\Gamma \left(\alpha_{i}\right)}\;+\;\overset{n}{\underset{i=1}{\sum }}(\alpha_{i}-1/2)\left\{\psi \left(\alpha_{i}\right)-\psi \left(\alpha_{0}\right)\right\}+\\ 1/2\sum_{j=1}^{N}\varepsilon_{i}^{-2}{\left(m_{j}-\lambda /\alpha_{0}\sum_{i=1}^{n}I_{ij}\alpha_{i}\right)}^{2}+\frac{1}{2}\sum_{i=1}^{n}\sum_{j=1}^{n}\left(\sum_{k=1}^{N}\frac{I_{ik}I_{jk}}{\varepsilon_{k}{}^{2}}\right)\frac{\alpha_{i}\left(\alpha_{0}-\alpha_{i}\right)\delta_{ij}-\alpha_{i}\alpha_{j}\left(1-\delta_{ij}\right)}{\alpha_{0}{}^{2}\left(\alpha_{0}+1\right)} \end{array}$$ (16)

where *δ*_*ij*_ is Kronecker delta function, *ψ*(·) is digamma function and *λ* is a scaling factor between experimental and ensemble averaged inferred measurements calculated according to the formula described in Svergun et al. [69]:

$$\lambda \;=\;\frac{\sum_{j\;=\;1}^{N}{\varepsilon_{j}}^{-2}{\alpha_{0}}^{-1}m_{j}\sum_{i\;=\;1}^{n}I_{ij}\alpha_{i}}{\sum_{j\;=\;1}^{N}{\varepsilon_{j}}^{-2}({\alpha_{0}}^{-1}\sum_{i\;=\;1}^{n}I_{ij}\alpha_{i}{)}^{2}}$$ (17)

When the Rosetta energies are used in the inference, *L* function has the following form:

$$\begin{array}{r} L\left(\alpha ,S,U_{ref}\right)=\log\frac{\Gamma \left(\alpha_{0}\right)}{\Gamma \left(\beta_{0}\right)}+\overset{n}{\underset{i=1}{\sum }}\log\frac{\Gamma \left(\beta_{i}\right)}{\Gamma \left(\alpha_{i}\right)}\;+\;\overset{n}{\underset{i=1}{\sum }}(\alpha_{i}-\;\beta_{i})\left\{\psi \left(\alpha_{i}\right)-\psi \left(\alpha_{0}\right)\right\}+\\ 1/2\overset{N}{\underset{j=1}{\sum }}\varepsilon_{j}^{-2}{\left(m_{j}-\lambda /\alpha_{0}\overset{n}{\underset{i=1}{\sum }}I_{ij}\alpha_{i}\right)}^{2}+\frac{1}{2}\overset{n}{\underset{i=1}{\sum }}\overset{n}{\underset{j=1}{\sum }}\left(\overset{N}{\underset{k=1}{\sum }}\frac{I_{ik}I_{jk}}{\varepsilon_{k}{}^{2}}\right)\frac{\alpha_{i}\left(\alpha_{0}-\alpha_{i}\right)\delta_{ij}-\alpha_{i}\alpha_{j}\left(1-\delta_{ij}\right)}{\alpha_{0}{}^{2}\left(\alpha_{0}+1\right)} \end{array}$$ (18)

In each round of the model selection algorithm the *L* function is minimized for the current set of conformations *S* by identifying the optimal set of parameters *α*_*i*_ and *U*_*ref*_ (when Rosetta energies are available) using simulated annealing. After finding the optimal weights through the *α*_*i*_ parameters, the conformers with lowest weights are removed from the ensemble by applying a cut, *w*_*cut*_ (fixed at the start of the simulation, explicit values are provided in S1 Table), so that conformers with *w*_*i*_ < *w*_*cut*_ are culled from the set. This procedure is repeated, and the simulation stops when the *L* function does not improve in 10 iterations. In the case of SAXS-only data and SAXS with NMR chemical shifts we restart optimization several times, starting from the set of structures from previous run until the *L* function did not improve (see S1 Table). When running simulations with structural energies this was not necessary because the algorithm converged in a single run. Because of the stochastic nature of the algorithm the inferred ensemble may not always converge to the same set of structural models and population weights. We repeated entire procedure 2 to 4 times depending on the data type used in the inference to monitor convergence and selected solutions with the lowest *L*. We implemented VBI using openmp library allowing for parallel computation, which provides considerable speed up compare to original method by Fisher et al. [29].

### Complete Bayesian inference

Once the small subset of models has been selected using VBI, we determine corresponding population weights with complete Bayesian inference. We based CBI implementation on the Stan library—platform for statistical modeling and high-performance statistical computation [35]. The weights $\vec{w}$, scaling factor *λ* and parameter defining the shape of Boltzmann distribution *U*_*ref*_ are sampled using Markov Chain Monte Carlo (MCMC) simulations. In each run we performed 2000 simulations with No-U-Turn sampler [34] using 4 chains and 4 jobs. We monitored MCMC simulations by inspecting the effective sample size and split $\hat{R}$ parameter, which are diagnostics available directly from Stan. In addition to these metrics, we used a few statistics from stan_utility (https://github.com/betanalpha/jupyter_case_studies/blob/master/pystan_workflow/): trajectory tree depth, energy Bayesian fraction of missing information and posterior parameters divergence.

VBI and CBI were implemented with python and C++ and are available from: https://andre-lab.github.io/bioce/ as well as through web-server: https://andre-lab.github.io/bioce/webserver.html.

### Accurate model evidence calculation

In the case when model evidence was explicitly evaluated and not approximated we performed numerical integration of the integral from Eq 5:

$$\int f(\vec{m}|\vec{w},S)f(\vec{w}|S)d\vec{w}$$ (19)

This was calculated by determining the expectation value of the likelihood function $f(\vec{m}|\vec{w},S)$ evaluated on the weight values sampled from prior distribution $f(\vec{w}|S)$ (Dirichlet distribution).

### Scattering profiles and chemical shifts calculations from molecular models

We used the FoXS [21] program to calculate scattering profiles from atomic coordinates of conformers. In cases where experimental data was available scattering profiles were calculated for experimental *q* values, otherwise we used equally spaced *q* values ranging from 0 to 0.5 1/nm (default in FoXS). Scattering profiles calculated on experimental *q* values were subsequently descaled by dividing intensities with the c1 scaling parameter (returned by FoXS) to have equally scaled intensities for the Bayesian inference. To predict NMR chemical shift data *C*_*ij*_ and their uncertainties *ε*_CS_ from the set of structural models we used the SHIFTX2 program [70]. Python scripts for generating scattering profiles and chemical shift data and converting them to the required input format are available with the software.

### Generation of structural models

To generate a library of energetically reasonable conformers of ΔmC2 and CaM we developed a sampling protocol in Rosetta macromolecular modeling package [71]. The protocol samples torsion angles in the linker segment using Monte Carlo simulations (1000 iterations) and subsequently repacks side chains. The linker modeling was followed by all atom energy refinement of the linker segment and the neighboring residues with fast relax protocol [72]. Around 10 000 models were generated by this procedure and the 1000 lowest energy conformers constituted the lowest energy structural library.

### Model selection with structural energies

In order to demonstrate that presence of low energy conformer does not considerably bias simulations towards Boltzmann weights, we used the Rosetta Relax protocol to optimize energy of one of the ΔmC2 models. Constraints on atomic coordinates were introduced to ensure that model did not substantially deviate from its starting conformation so that the scattering pattern of the energy-refined model is highly similar.

### SAXS and NMR chemical shift experimental data

NMR chemical shifts measurements for CaM were described in [73] and the data was obtained from Biological Magnetic Resonance Data Bank (BMRB Entry 547). This data was recorded for CaM from Drosophila, which differs from human CaM in three amino acid positions: Y99F, N143T, and T136S. We excluded these three substitutions in our simulations by omitting them in experimental and predicted chemical shift data. SEC-SAXS data for CaM are deposited in the SASBDB (https://www.sasbdb.org/), identifier SASDCQ2, and fully described in [14] an open access article for which the CaM data are publicy available under the uniform resource identifier https://creativecommons.org/licenses/by/2.0/uk/legalcode. SAXS data for ΔmC2 are deposited in SASBDB (identifier SASDDD9), while NMR chemical shift data was taken from [37].

### Inferring conformational ensembles with MultiFoXS and EOM

The web version of MultiFoXS (https://modbase.compbio.ucsf.edu/multifoxs/ [21]) and the ATSAS on-line version of EOM (https://www.embl-hamburg.de/biosaxs/atsas-online/ [67]) were used to obtain the multi-state and ensemble optimization modelling results, respectively, for CaM and ΔmC2 shown in S5 Table. The crystal structure coordinates of CaM (PDB:1CLL) and Model 1 from the NMR ensemble for ΔmC2 were the starting structures (PDB:5K6P). In the case of CaM the 3 missing N terminal amino acids (Ala1, Gln2, Asp3) from the crystal structure and the flexible linker (Lys77, Asp78, Thr79, Asp80, Ser81) were assigned unknown structure. In the case of ΔmC2 the 7-amino acid flexible linker (Arg356, Arg357, Asp358, Glu359, Lys360, Lys361, Ser362) was assigned unknown structure. MultiFoXS generates structures for the unknown regions that have correct stereochemistry, while for EOM the random coil option was chosen to model the missing amino acids. The SAXS data used for modelling were for CaM SASBDB ID SASDCQ2, *q* = range 0.0066–0.3104 Å^-1^, and for ΔmC2 SASBDB ID SASDDD9.

### Model selection with various information content

The amount of structural information covered by SAXS experimental data was assessed using the BayesApp program [33]. We included all data points in the analysis and used default input parameters.

### Radius of gyration distributions

The radius of gyration for individual models was calculated using CRYSOL program from ATSAS package [74].

## Supporting information

S1 Fig Inference of ensembles with Variational Bayesian model selection. Synthetic data were generated for an ensemble of 5 conformers (models 1–5) with population weights of 0.1, 0.15, 0.2, 0.25 and 0.30, and added synthetic noise. The starting model set for the inference included 100 models. The weights for the 5 conformers in the model ensemble are plotted as a function of iteration number in the ensemble selection algorithm. The posterior weights from the process are 0.1, 0.15, 0.2, 0.25 and 0.30, which exactly match priest values.
(TIF)

S2 Fig Inference with the energy prior. Synthetic data was generated for five conformers (model 1–5) with weights of 0.1, 0.15, 0.2, 0.25 and 0.3 and added experimental noise. An energy prior with Boltzmann weights of 0.09, 0.29, 0.20, 0.18 and 0.24 was employed in the simulation. The rmsd relative to the assigned population weights (red) and the Boltzmann weights (blue) as a function of increasing noise.
(TIF)

S3 Fig Inference with and without the energy prior from the structural library containing energy refined conformers. Synthetic data was generated for the 5 lowest energy conformers (model 1–5) in a library of 100 members. The ensemble was simulated by assigning equal weights of 0.2 to each of the 5 conformers and adding experimental noise. An energy prior was used based on Rosetta energies of the selected 5 conformers (-135.2, -140.0, -126.7, -125.5, -124.0). The lowest energy model (energy of -140.0) was further refined using Rosetta software suite. The resulting model has energy of -178.9 and was added to the library of structural models. Therefore, simulations were performed with the library of 101 members. Plot of the number of recovered members of the simulated ensemble as a function of noise with (red) and without (blue) the energy prior as a function of increasing simulated noise.
(TIF)

S4 Fig Posterior predictive check validates underlying statistical model. The set of curves (orange, red, and green on main plot and inset) generated from statistical model comprising prior distribution and likelihood function as used in simulation gives almost perfect agreement with experimental data (blue curve). There are only small variations are noted in high q region (inset). The data predicted from Cauchy distribution (purple curve) gives considerably worse fit to experimental data.
(TIF)

S5 Fig Radius of gyration distributions of 10000 models generated with Rosetta Monte Carlo simulations and 1000 lowest energy models for calmodulin (A and B) and ΔmC2 (C and D). (TIF)

S1 Table Variational Bayesian inference details for calmodulin and ΔmC2. (DOCX)

S2 Table Complete Bayesian inference of calmodulin and ΔmC2 from SAXS, NMR chemical shift data and with or without structural energies. (DOCX)

S3 Table Three best matching calmodulin ensemble models (from Bayesian inference) with CaM complexes available from Protein Data Bank (PDB) as listed bellow^*a*^. (DOCX)

S4 Table Population weights, fit to SAXS data and JSD values from CBI following VBI using SAXS only data. (DOCX)

S5 Table Bayesian, MultiFoxs and EOM ensembles comparison. Direct comparison can be performed for SAXS data only, however other Bayesian inference scenarios are also listed for reference.
(DOCX)
