# Supplementary figures and images for: Bayesian inference of protein conformational ensembles from limited structural data
Source: PLoS Comput Biol. 2018 Dec 17;14(12):e1006641. doi: 10.1371/journal.pcbi.1006641 (PMC6312354; doi:10.1371/journal.pcbi.1006641)

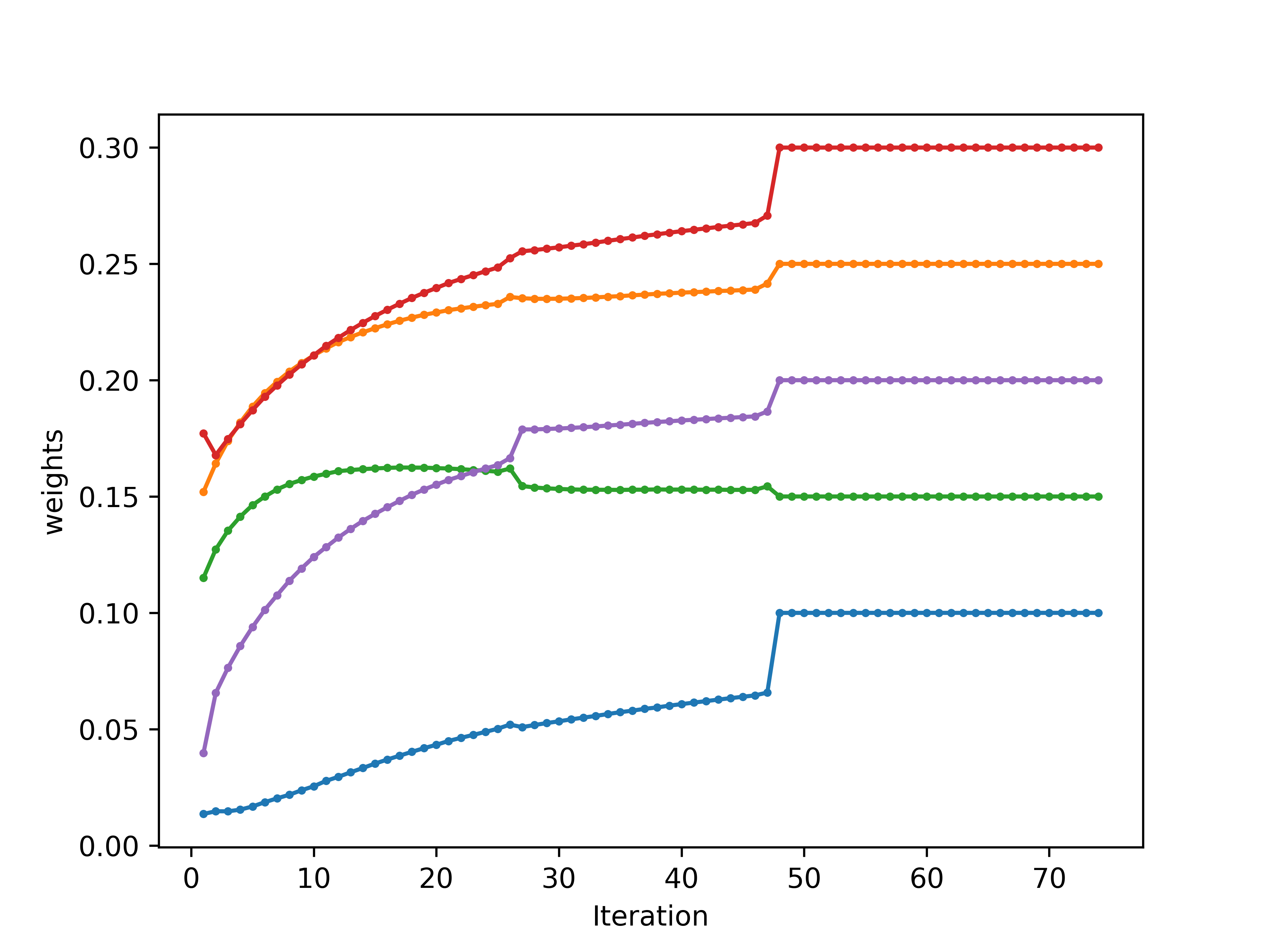

Supplement: S1 Fig — Synthetic data were generated for an ensemble of 5 conformers (models 1–5) with population weights of 0.1, 0.15, 0.2, 0.25 and 0.30, and added synthetic noise. The starting model set for the inference included 100 models. The weights for the 5 conformers in the model ensemble are plotted as a function of iteration number in the ensemble selection algorithm. The posterior weights from the process are 0.1, 0.15, 0.2, 0.25 and 0.30, which exactly match priest values. (TIF) [file pcbi.1006641.s001.tif]

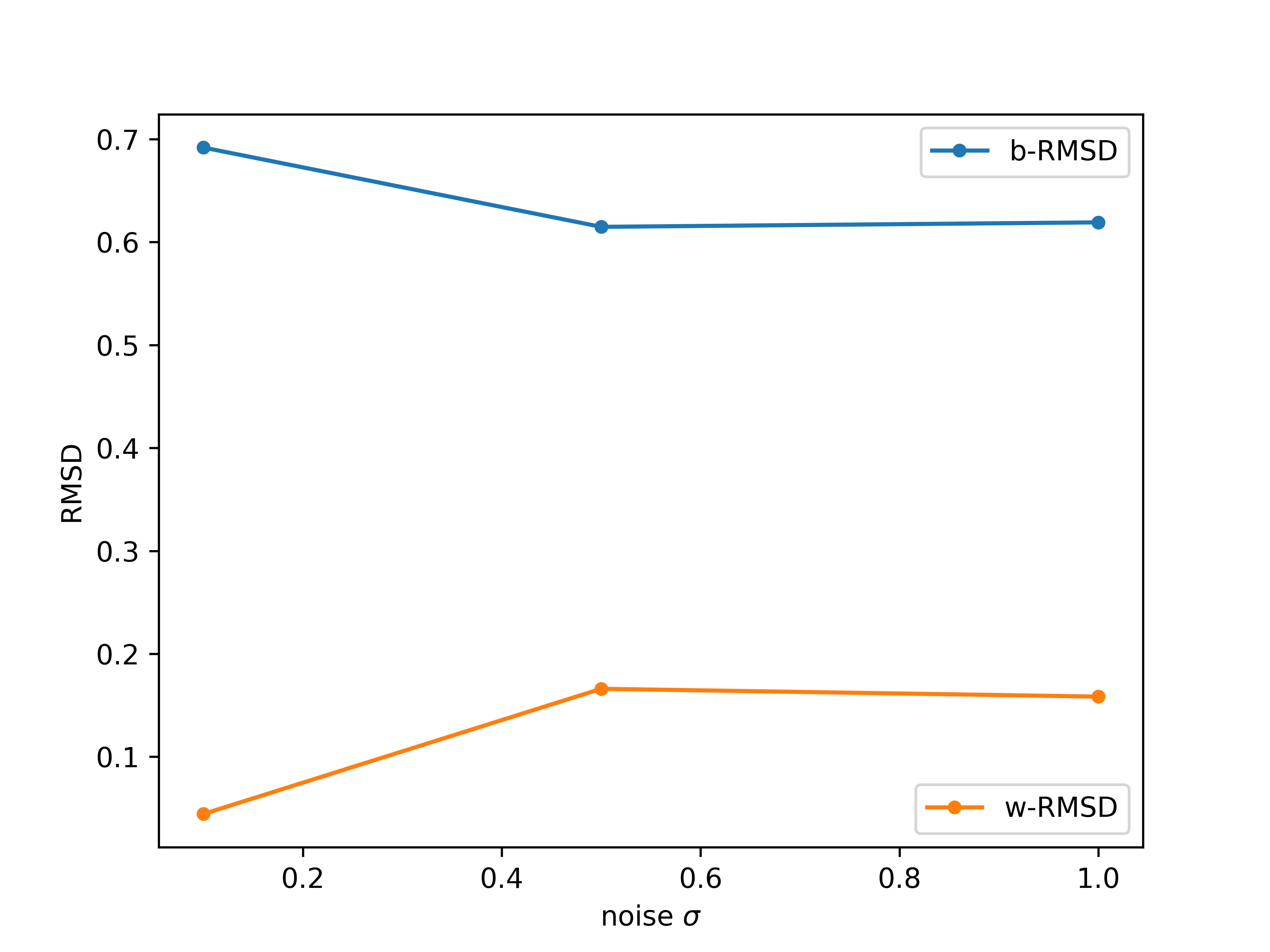

Supplement: S2 Fig — Synthetic data was generated for five conformers (model 1–5) with weights of 0.1, 0.15, 0.2, 0.25 and 0.3 and added experimental noise. An energy prior with Boltzmann weights of 0.09, 0.29, 0.20, 0.18 and 0.24 was employed in the simulation. The rmsd relative to the assigned population weights (red) and the Boltzmann weights (blue) as a function of increasing noise. (TIF) [file pcbi.1006641.s002.tif]

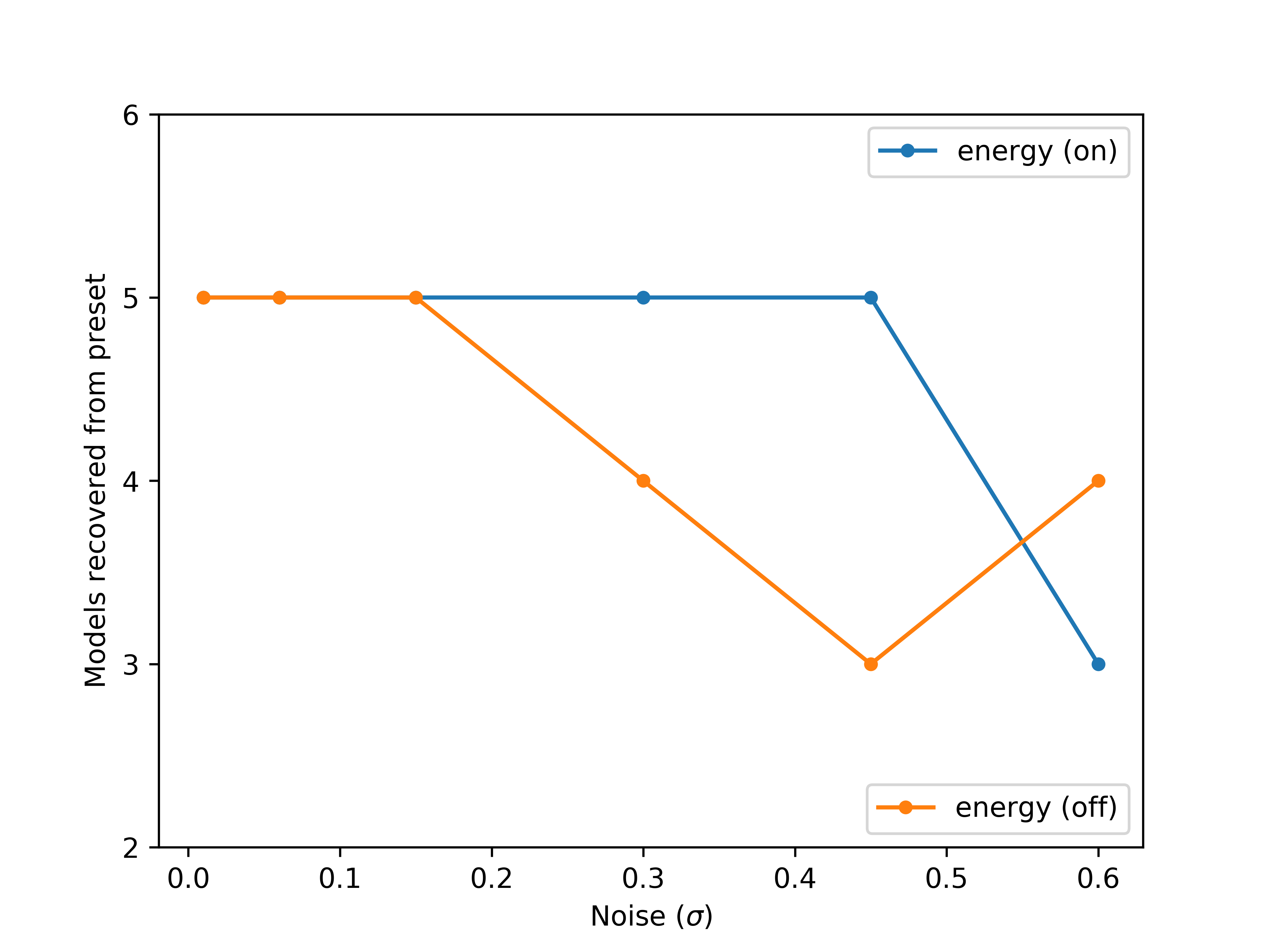

Supplement: S3 Fig — Synthetic data was generated for the 5 lowest energy conformers (model 1–5) in a library of 100 members. The ensemble was simulated by assigning equal weights of 0.2 to each of the 5 conformers and adding experimental noise. An energy prior was used based on Rosetta energies of the selected 5 conformers (-135.2, -140.0, -126.7, -125.5, -124.0). The lowest energy model (energy of -140.0) was further refined using Rosetta software suite. The resulting model has energy of -178.9 and was added to the library of structural models. Therefore, simulations were performed with the library of 101 members. Plot of the number of recovered members of the simulated ensemble as a function of noise with (red) and without (blue) the energy prior as a function of increasing simulated noise. (TIF) [file pcbi.1006641.s003.tif]

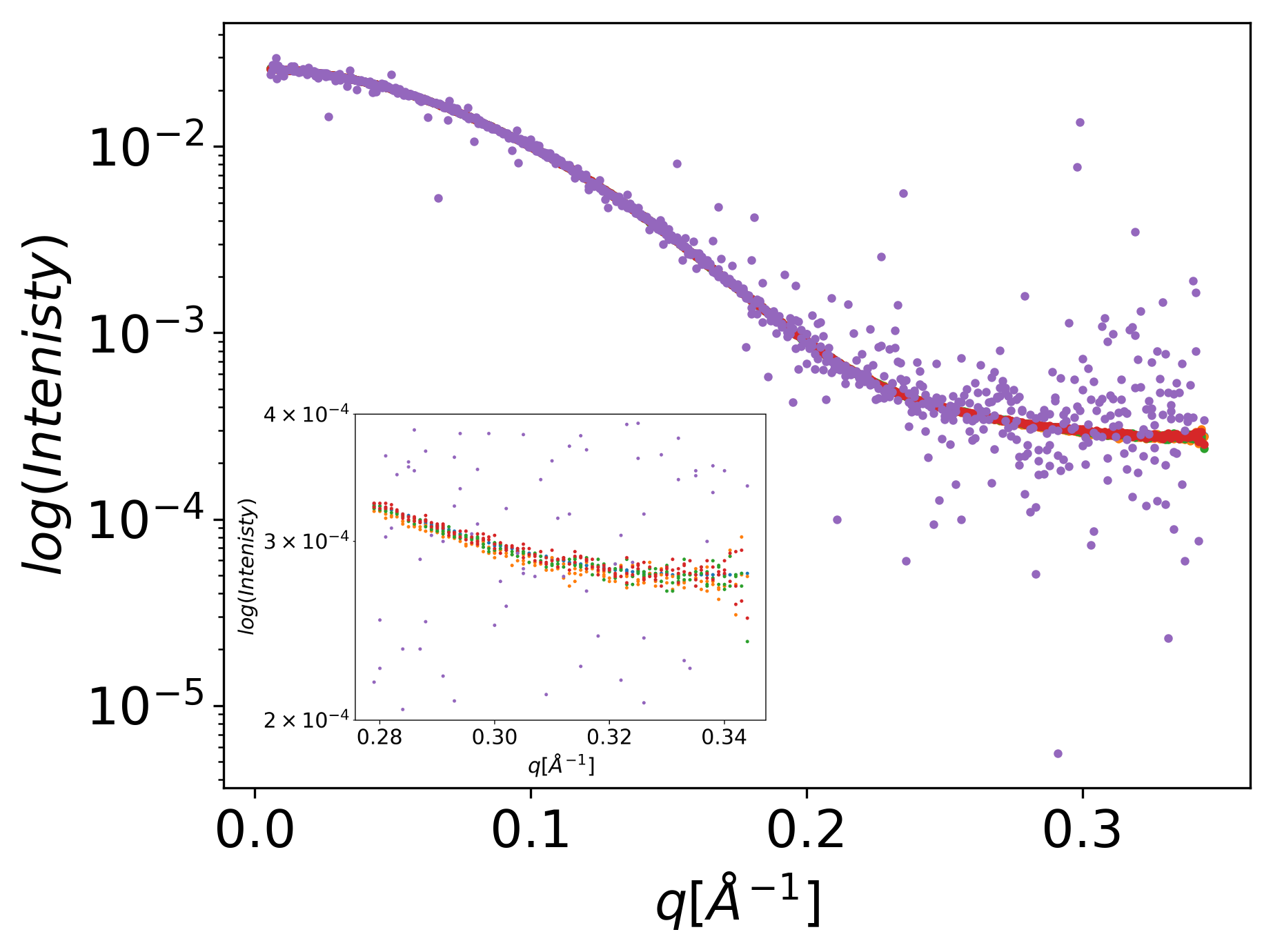

Supplement: S4 Fig — The set of curves (orange, red, and green on main plot and inset) generated from statistical model comprising prior distribution and likelihood function as used in simulation gives almost perfect agreement with experimental data (blue curve). There are only small variations are noted in high q region (inset). The data predicted from Cauchy distribution (purple curve) gives considerably worse fit to experimental data. (TIF) [file pcbi.1006641.s004.tif]

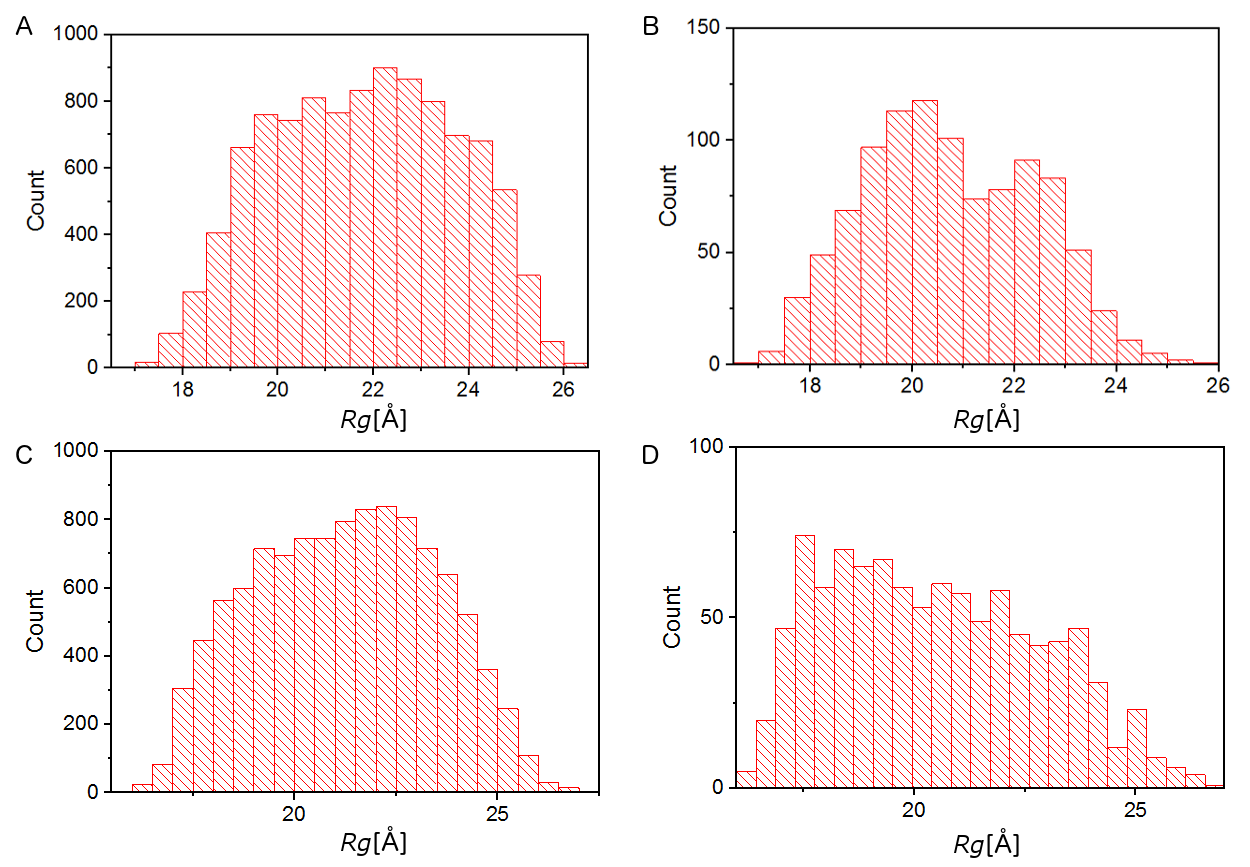

Supplement: S5 Fig — (TIF) [file pcbi.1006641.s005.tif]
