# Supplementary material for: Bayesian inference of protein conformational ensembles from limited structural data
Source: PLoS Comput Biol. 2018 Dec 17;14(12):e1006641. doi: 10.1371/journal.pcbi.1006641 (PMC6312354; doi:10.1371/journal.pcbi.1006641)
Supplement: S1 Table — (DOCX) [file pcbi.1006641.s006.docx]

**S1 Table.** Variational Bayesian inference details for calmodulin and ΔmC2

| Data | Iter. | Starting models | End models | w*_cut_* | χ^2^ |
| --- | --- | --- | --- | --- | --- |
| Calmodulin |  |  |  |  |  |
| SAXS | 1 | 1004^a^ | 459 | 0.0003 |  |
|  | 2 | 459 | 51 | 0.001 |  |
|  | 3 | 51 | 4 | 0.02 | 0.83 |
| SAXS+ structural energies | 1 | 1004 | 3 | 0.0001 | 0.87 |
| SAXS + CS | 1 | 1004 | 4 | 0.0001 | 0.81 |
| SAXS + CS + structural energies | 1 | 1004 | 3 | 0.0001 | 0.87 |
|  |  |  |  |  |  |
| ΔmC2 |  |  |  |  |  |
| SAXS | 1 | 1006^a^ | 540 | 0.0004 |  |
|  | 2 | 540 | 152 | 0.0007 |  |
|  | 3 | 152 | 5 | 0.006 | 3.77 |
| SAXS+ structural energies | 1 | 1006 | 3 | 0.0002 | 3.55 |
| SAXS + CS | 1 | 1006 | 419 | 0.0004 |  |
|  | 2 | 419 | 5 | 0.002 | 3.81 |
| SAXS + CS + structural energies | 1 | 1006 | 4 | 0.0004 | 3.56 |

*a*)1004 and 1006 models in the structural libraries are due to the fact that 5 and 7 models respectively have same energy and therefore they were selected when energy filter for 1000 lowest energy conformers was applied.
