# Supplementary material for: Bayesian inference of protein conformational ensembles from limited structural data
Source: PLoS Comput Biol. 2018 Dec 17;14(12):e1006641. doi: 10.1371/journal.pcbi.1006641 (PMC6312354; doi:10.1371/journal.pcbi.1006641)
Supplement: S2 Table — (DOCX) [file pcbi.1006641.s007.docx]

**S2 Table.** Complete Bayesian inference of calmodulin and ΔmC2 from SAXS, NMR chemical shift data and with or without structural energies.

|  | Ensemble members weights and (Rg [Å]) | | | | |
| --- | --- | --- | --- | --- | --- |
| Calmodulin |  | | | | |
| SAXS (variant 1) | 4*^a^* | 1 | 2 | 3 |  |
|  | 0.12±0.02 (20.6) | 0.02±0.01 (23.0) | 0.69±0.02 (22.6) | 0.17±0.04 (21.1) |  |
| SAXS + Rosetta energies (variant 2) | 6 | 7 | 5 |  |  |
|  | 0.06±0.01 (26.0) | 0.4±0.05 (21.8) | 0.54±0.06 (22.6) |  |  |
| SAXS + CS (variant 3) | 7 | 8 | 9 | 10 |  |
|  | 0.12±0.05 (22.3) | 0.45±0.04 (22.7) | 0.16±0.03 (22.6) | 0.26±0.02 (21.5) |  |
| SAXS + CS + Rosetta energies (variant 4) | 12 | 13 | 11 |  |  |
|  | 0.24±0.04 (21.8) | 0.19±0.03 (21.8) | 0.57±0.02 (22.6) |  |  |
|  | | | | | |
| ΔmC2 |  | | | | |
| SAXS (variant 1) | 6*^b^* | 2 | 1 | 4 | 7 |
|  | 0.21±0.03 (19.3) | 0.05±0.02 (24.0) | 0.17±0.02 (27.0) | 0.43±0.04 (18.9) | 0.15±0.02 (16.9) |
| SAXS+ Rosetta energies (variant 2) | 2 | 3 | 1 |  |  |
|  | 0.53±0.02 (24.0) | 0.42±0.01 (18.1) | 0.05±0.02 (27.0) |  |  |
| SAXS + CS (variant 3) | 1 | 9 | 5 | 8 | 4 |
|  | 0.18±0.01 (27.0) | 0.19±0.01 (17.8) | 0.44±0.05 (19.5) | 0.03±0.03 (18.9) | 0.18±0.07 (18.9) |
| SAXS + CS + Rosetta energies (variant 4) | 2 | 3 | 1 | 5 |  |
|  | 0.26±0.04 (24.0) | 0.26±0.02 (18.1) | 0.16±0.02 (27.0) | 0.32±0.05 (19.5) |  |

*a*) model numbering follows Figure 2 in main text

*b*) model numbering follows Figure 3 in main text
