# Supplementary material for: Bayesian inference of protein conformational ensembles from limited structural data
Source: PLoS Comput Biol. 2018 Dec 17;14(12):e1006641. doi: 10.1371/journal.pcbi.1006641 (PMC6312354; doi:10.1371/journal.pcbi.1006641)
Supplement: S3 Table — (DOCX) [file pcbi.1006641.s008.docx]

**S3 Table.** Three best matching calmodulin ensemble models (from Bayesian inference) with CaM complexes available from Protein Data Bank (PDB) as listed bellow*^a^*.

| Inference scenario | Model | PDB | RMSD [Å] |
| --- | --- | --- | --- |
| SAXS | 1*^b^* | 2KDU.pdb, chain A (#1.18) | 5.1 |
|  | 2 | 1CFF.pdb, chain A (#1.23) | 5.7 |
|  | 3 | 2L53.pdb, chain A (#1.15) | 6.7 |
|  |  | | |
| SAXS + energies | 5 | 1CFF.pdb, chain A (#1.8) | 4.6 |
|  | 6 | 4DJC.pdb, chain A (#1) | 4.6 |
|  | 7 | 2KDU.pdb, chain A (#1.3) | 4.8 |
|  |  | | |
| SAXS + CS | 7 | 1CFF.pdb, chain A (#1.8) | 5.2 |
|  | 8 | 1CFF.pdb, chain A (#1.14) | 6.8 |
|  | 10 | 1CFF.pdb, chain A (#1.25) | 7.3 |
|  |  | | |
| SAXS + CS + energies | 11 | 1CFF.pdb, chain A (#1.8) | 6.2 |
|  | 12 | 2L53.pdb, chain A (#1.19) | 6.6 |
|  | 13 | 2KDU.pdb, chain A (#1.1) | 6.4 |

*a)* 72 calmodulin complexes were used in comparison (PDB ids: 1CDL, 1CDM, 1CFF, 1CKK, 1CM1, 1G4Y, 1IQ5, 1IWQ, 1K90, 1L7Z, 1LVC, 1MXE, 1NIW, 1NWD, 1PK0, 1QS7, 1QTX, 1QX7, 1S26, 1SK6, 1SY9, 1WRZ, 1XFU, 2BBM, 2BCX, 2BE6, 2F2O, 2F3Y, 2FOT, 2HQW, 2IX7, 2JZI, 2K0F, 2KDU, 2KNE, 2L1W, 2L53, 2L7L, 2LGF, 2LHI, 2LL6, 2O5G, 2O60, 2R28, 2VAY, 2W73, 2WEL, 2XOG, 2Y4V, 2YGG, 3BXK, 3BXL, 3BYA, 3DVE, 3DVJ, 3DVK, 3DVM, 3EWT, 3EWV, 3G43, 3GOF, 3GP2, 3HR4, 3OXQ, 3SJQ, 3SUI, 4AQR, 4DCK, 4DJC, 4DS7, 4EHQ, 4G27).

*b)* Model numbering corresponds to figure 2 in the main text.
