# Supplementary material for: Bayesian inference of protein conformational ensembles from limited structural data
Source: PLoS Comput Biol. 2018 Dec 17;14(12):e1006641. doi: 10.1371/journal.pcbi.1006641 (PMC6312354; doi:10.1371/journal.pcbi.1006641)
Supplement: S4 Table — (DOCX) [file pcbi.1006641.s009.docx]

**S4 Table.** Population weights, fit to SAXS data and JSD values from CBI following VBI using SAXS only data.

|  | **CaM** | | | | **ΔmC2** | | | |
| --- | --- | --- | --- | --- | --- | --- | --- | --- |
|  | SAXS^a^ | SAXS+energies | SAXS+CS | SAXS+CS+  energies | SAXS^a^ | SAXS+energies | SAXS+CS | SAXS+CS+  energies |
| *w_1_* | 0.12 | 0.12 | 0.11 | 0.11 | 0.21 | 0.21 | 0.19 | 0.2 |
| *w_2_* | 0.02 | 0.02 | 0.02 | 0.02 | 0.04 | 0 | 0.05 | 0 |
| *w_3_* | 0.69 | 0.69 | 0.69 | 0.69 | 0.17 | 0.21 | 0.17 | 0.21 |
| *w_4_* | 0.17 | 0.17 | 0.18 | 0.18 | 0.43 | 0.48 | 0.44 | 0.49 |
| w_5_ |  |  |  |  | 0.15 | 0.11 | 0.15 | 0.11 |
| χ^2^ | 0.83 | 0.83 | 0.83 | 0.83 | 3.77 | 3.78 | 3.77 | 3.78 |
| JSD | 0.07 | 0.07 | 0.07 | 0.07 | 0.07 | 0.05 | 0.07 | 0.04 |

1. SAXS ensemble is kept here for comparison
