# Supplementary material for: Bayesian inference of protein conformational ensembles from limited structural data
Source: PLoS Comput Biol. 2018 Dec 17;14(12):e1006641. doi: 10.1371/journal.pcbi.1006641 (PMC6312354; doi:10.1371/journal.pcbi.1006641)
Supplement: S5 Table — Direct comparison can be performed for SAXS data only, however other Bayesian inference scenarios are also listed for reference. (DOCX) [file pcbi.1006641.s010.docx]

**S5 Table.** Bayesian, MultiFoxs and EOM ensembles comparison. Direct comparison can be performed for SAXS data only, however other Bayesian inference scenarios are also listed for reference.

| Calmodulin | | | | | | | | | | | | |
| --- | --- | --- | --- | --- | --- | --- | --- | --- | --- | --- | --- | --- |
|  | MultiFoxS | | EOM | | Bayesian inference 1  (SAXS) | | Bayesian inference 2 (SAXS+energies) | | Baysian inference 3 (SAXS+CS) | | Bayesian inference 4 (SAXS+CS+energies) | |
|  | 2 models | | 9 models | | 4 models | | 3 models | | 4 models | | 3 models | |
|  | *R_g_* range (Å) | wght. | *R_g_* range (Å) | wght. | *R_g_* range (Å) | wght. | *R_g_* range (Å) | wght. | *R_g_* range (Å) | wght. | *R_g_* range (Å) | wght. |
|  |  |  | 25.2 | 0.08 |  |  | 26.0 | 0.06 |  |  |  |  |
|  | 22.3 | 0.7 | 22.3-23.8 | 0.50 | 22.3-22.6 | 0.71 | 22.6 | 0.54 | 22.3-22.7 | 0.74 | 22.7 | 0.57 |
|  | 19.5 | 0.3 | 18.9-21.0 | 0.42 | 20.6-21.0 | 0.29 | 21.8 | 0.40 | 21.5 | 0.26 | 21.8 | 0.43 |
| Quality of fit | | | | | | | | | | | | |
| χ^2^ *^a^* | 0.79 | | 0.81 | | 0.84 | | 0.86 | | 0.82 | | 0.87 | |
| P-value (size; locus, Å^-1^) | 0.53 (8; 0.029) | | 0.79 (8; 0.0818) | | 0.79 (8;0.0293) | | 0.79 (8, 0.0293) | | 0.96 (7; 0.2799) | | 0.534 (9; 0.2476) | |
| ΔmC2 | | | | | | | | | | | | |
|  | MultiFoxS | | EOM | | Bayesian inference 1  (SAXS) | | Bayesian inference 2 (SAXS+energies) | | Baysian inference 3 (SAXS+CS) | | Bayesian inference 4 (SAXS+CS+energies) | |
|  | 2 models | | 15 models | | 5 models | | 3 models | | 5 models | | 4 members | |
|  | *R_g_* range (Å) | wght. | *R_g_* range (Å) | wght. | *R_g_* range (Å) | wght. | *R_g_* range (Å) | wght. | *R_g_* range (Å) | wght. | *R_g_* range (Å) | wght. |
|  |  |  |  |  | 27.0 | 0.16 | 27.0 | 0.05 | 27.0 | 0.18 | 27.0 | 0.16 |
|  | 23.1 | 0.25 | 22.5-25.2 | 0.23 | 24.0 | 0.05 | 24.0 | 0.53 |  |  | 24.0 | 0.26 |
|  | 17.5 | 0.75 | 16.7-20.5 | 0.77 | 16.9-19.3 | 0.80 | 18.1 | 0.42 | 17.8-19.5 | 0.84 | 18.1-19.5 | 0.58 |
| Quality of fit | | | | | | | | | | | | |
| χ^2^ *^a^* | 3.59 | | 3.58 | | 3.78 | | 3.57 | | 3.82 | | 3.60 | |
| P-value (size; locus, Å^-1^) | 0.82 (9; 0.1616) | | 0.97 (8, 0.1616) | | 0.19 (12; 0.3122) | | 0.81 (9; 0.1616) | | 0.19 (12; 0.3122) | | 0.81 (9; 0.1616) | |

a) χ^2^ values are all from primusqt data comparison.
